# A plastid phylogenomic framework for the palm family (Arecaceae)

**DOI:** 10.1186/s12915-023-01544-y

**Published:** 2023-03-08

**Authors:** Gang Yao, Yu-Qu Zhang, Craig Barrett, Bine Xue, Sidonie Bellot, William J. Baker, Xue-Jun Ge

**Affiliations:** 1 College of Forestry and Landscape Architecture, South China Agricultural University, Guangzhou, 510642 China; 2 Key Laboratory of Plant Resources Conservation and Sustainable Utilization, and Guangdong Provincial Key Laboratory of Applied Botany, South China Botanical Garden, Chinese Academy of Sciences, Guangzhou, China; 3 Present Address: College of Pharmacy, Shaanxi University of Chinese Medicine, Xi’an, China; 4 Department of Biology, West Virginia University, Morgantown, WV USA; 5 College of Horticulture and Landscape Architecture, Zhongkai University of Agriculture and Engineering, Guangzhou, 510225 China; 6 Royal Botanic Gardens, Kew, TW9 3AB Surrey UK; 7 Center of Conservation Biology, Core Botanical Gardens, Chinese Academy of Sciences, Guangzhou, China

**Keywords:** Arecaceae, Nuclear-plastid discordance, Palmae, Plastome, Phylogenomics

## Abstract

**Background:**

Over the past decade, phylogenomics has greatly advanced our knowledge of angiosperm evolution. However, phylogenomic studies of large angiosperm families with complete species or genus-level sampling are still lacking. The palms, Arecaceae, are a large family with ca. 181 genera and 2600 species and are important components of tropical rainforests bearing great cultural and economic significance. Taxonomy and phylogeny of the family have been extensively investigated by a series of molecular phylogenetic studies in the last two decades. Nevertheless, some phylogenetic relationships within the family are not yet well-resolved, especially at the tribal and generic levels, with consequent impacts for downstream research.

**Results:**

Plastomes of 182 palm species representing 111 genera were newly sequenced. Combining these with previously published plastid DNA data, we were able to sample 98% of palm genera and conduct a plastid phylogenomic investigation of the family. Maximum likelihood analyses yielded a robustly supported phylogenetic hypothesis. Phylogenetic relationships among all five palm subfamilies and 28 tribes were well-resolved, and most inter-generic phylogenetic relationships were also resolved with strong support.

**Conclusions:**

The inclusion of nearly complete generic-level sampling coupled with nearly complete plastid genomes strengthened our understanding of plastid-based relationships of the palms. This comprehensive plastid genome dataset complements a growing body of nuclear genomic data. Together, these datasets form a novel phylogenomic baseline for the palms and an increasingly robust framework for future comparative biological studies of this exceptionally important plant family.

**Supplementary Information:**

The online version contains supplementary material available at 10.1186/s12915-023-01544-y.

## Background

Over the past decade, enormous progress in phylogenomics has been made in clarifying many recalcitrant relationships among angiosperms [1–5]. However, disentangling the relationships among angiosperm lineages that have undergone rapid radiations remains a major challenge [1, 4–7] and is often complicated by the existence of long branches [8], hybridization [9], incomplete lineage sorting (ILS) [4, 10], polyploidization [9, 11], and lateral gene transfer [12]. The importance of broad taxon sampling in improving the accuracy of phylogenetic inference has been highlighted by many authors [13–16], since including more taxa can improve the detection of multiple substitutions [17, 18] and thus may be helpful to alleviate the influence of systematic error, such as “long-branch attraction” (LBA) [14, 19]. On the other hand, including more genomic data is also known to be helpful in improving phylogenetic inference, because this increases phylogenetic signal and may also reveal conflicting gene histories that can shed light on evolutionary history. For instance, comparing nuclear and plastid trees can help detect cases of past or recent hybridization [20, 21]. Previous phylogenetic studies of large angiosperm families based on the analysis of one or a few short genetic loci have included comprehensive or even complete generic sampling, but some key relationships remained unresolved or weakly supported, such as in the grass family Poaceae [22], the mint family Lamiaceae [23], and the palm family Arecaceae [24, 25]. An increasing number of phylogenomic studies (i.e., usually based on hundreds of generic regions) have been conducted recently to improve phylogenetic resolution in large angiosperm families, such as in Apiaceae [26], Apocynaceae [27], Asteraceae [28], Brassicaceae [29], Cucurbitaceae [30], Fabaceae [31, 32], Gesneriaceae [33], Lamiaceae [34], Orchidaceae [35], Poaceae [36], and Rosaceae [37, 38]. However, in these studies, sampling was mostly focused on the subfamily and/or tribal levels. Phylogenomic analyses with a comprehensive generic-level sampling of large angiosperm families remain rare and thus urgently needed to better understand the angiosperm tree of life.

Arecaceae, the palms, are an iconic large family of flowering plants [25, 39]. They represent one of the most diverse monocot families with ca. 181 genera and 2600 species [40–42], which are classified currently into five subfamilies and 28 tribes based on the results of extensive molecular phylogenetic studies [40]. Members of the palm family can be readily identified by their “woody” type growth, with primary thickening of their vascular tissues, plicate leaves with unique development, and inflorescences subtended by adaxial two-keeled bracts [43]. The palms exhibit a remarkable degree of morphological variation and over 90% of palm species are restricted to tropical rainforests, thus making Arecaceae an important model system to study the evolutionary history of tropical biodiversity [44, 45]. Additionally, the palm family includes many economically important species as hundreds of species are used by communities throughout the world for food, materials, medicine, and other uses [46]. Examples include the betelnut (*Areca catechu* L.), coconut (*Cocos nucifera* L.), date palm (*Phoenix dactylifera* L.), oil palm (*Elaeis guineensis* Jacq.), sago palm (*Metroxylon* Rottb. spp.), and wax palm (*Copernicia cerifera* (Arruda) Mart.). Furthermore, many palm species are economically important in the horticulture industry, being widely used in gardens and as ornamental trees and shrubs in landscaping [43].

As an ecologically and economically important angiosperm family, the palms have attracted the attention of many botanists and evolutionary biologists [25, 39, 41–51]. Molecular phylogenetic studies have greatly advanced our knowledge of the taxonomy and phylogeny of palms. A series of phylogenetic studies strongly supported the monophyly of Arecaceae and its affiliation within the commelinid monocots [6, 47, 52, 53]. However, as recovered in previous phylogenetic studies [25, 39, 47], diversification along the backbone of Arecaceae is complicated and disentangling palm tribal-level relationships is challenging. Early phylogenetic studies based on limited taxon sampling and a few plastid and nuclear DNA regions recovered several major clades within Arecaceae, but relationships among these clades were incompletely resolved [54–56]. Asmussen et al. [57] reconstructed the phylogenetic relationships of 161 palm genera based on the analysis of four plastid DNA regions (*matK*, *rbcL*, *rps16* intron, *trnL-F*) and recognized five subfamilies, viz., Arecoideae, Calamoideae, Ceroxyloideae, Coryphoideae, and Nypoideae. The first complete generic-level phylogenetic study of Arecaceae was conducted based on analyses of multiple plastid and nuclear loci, morphology, and restriction profiling [24]. In this study, which was central to the establishment of the formal phylogenetic classification [24], the monophyly of all the five subfamilies was strongly supported with increases in support values for many nodes compared with previous studies. Nevertheless, relationships among a number of tribes and genera were still weakly supported and some of the tribes and genera were even clustered in polytomies, especially among members of the subfamily Arecoideae, which represents over 60% of the generic diversity and nearly 55% of the species diversity in the family [24]. In a phylogenetic analysis of the palms using an all-evidence supertree approach based on ca. 35.5% species-level taxon sampling and 13 DNA markers (9 plastid and 4 nuclear regions), low support was obtained for relationships among some tribes and subtribes as well [25]. Seventy-four plastid genomes of 54 palm genera representing five subfamilies and 19 tribes were analyzed by Chen et al. [58], and relationships among all of them were mostly resolved with high support, but multiple tribes within the largest subfamily Arecoideae were not included. In a recent phylogenomic study of 2333 angiosperm genera based on an analysis of 353 nuclear genes, 67 genera representing 26 palm tribes and all the five palm subfamilies were included, the subfamily-level relationships and most tribal-level relationships were highly supported except some tribal-level relationships within Arecoideae were resolved with weak support [59]. An updated version of this phylogenetic tree is now available online via the Kew Tree of Life Explorer (KTLE) [60] and includes 179 palm genera. However, some tribal-level relationships in Arecoideae, Ceroxyloideae, and Coryphoideae were poorly resolved in this phylogenetic tree. Multiple phylogenomic studies based on large-scale nuclear or plastid genomic data were also conducted recently in different palm lineages, such as in Arecoideae [39, 48], Dypsidinae [41], Calamoideae [49], Lepidocaryeae [61], Phytelepheae [62], Geonomateae [63], *Brahea* Mart. [64], and *Raphia* P. Beauv. [65]. All these studies continuously improved our understanding of the evolution of the family. Nevertheless, in terms of phylogenetic relationships among tribes, subtribes, and genera, many nodes are still unresolved or weakly supported [24, 25, 39, 48, 57, 59]. Especially along the backbone of the largest subfamily Arecoideae, phylogenetic relationships among the six tribes within the core arecoids (Areceae, Euterpeae, Geonomateae, Leopoldinieae, Manicarieae and Pelagodoxeae [66]) need to be further clarified. Additionally, about 17 palm genera from the two largest subfamilies Arecoideae and Coryphoideae remain unplaced at the subtribal level due to their unresolved phylogenetic placements [40].

Plastid genomes provide a large number of nucleotide sites for phylogenetic inference and can be sequenced from many samples at a lower cost than nuclear genome regions [5, 13, 34]. A plastome tree can be compared to a nuclear tree to provide complementary insights on lineage history, for instance by revealing cases of past hybridization only detectable through chloroplast capture [20, 21], or aspects related to maternal inheritance [67]. Thus, plastid phylogenomics have been adopted extensively in recent studies of plant lineages both at deep and shallow taxonomic levels, such as in liverworts [68], ferns [69, 70], gymnosperms [71], and angiosperms [5, 10, 13, 31, 34, 37, 72–74], providing critical insights into the recalcitrant phylogenetic relationships within these groups. In contrast with the continuously increasing body of nuclear-based palm phylogenomic studies [39, 41, 49, 59–63], a comprehensive generic-level plastid phylogenomic study of the family is still lacking [47, 58]. Here, we conducted a plastid phylogenomic analysis of palms based on a nearly complete generic-level sampling and aimed at (1) shedding new light on the relationships among tribes, subtribes, and genera of palms and (2) evaluating how plastome and nuclear histories differ across the family.

## Results

### Plastome and dataset characteristics

Plastomes of 210 individuals representing 182 palm species and 111 genera were newly sequenced in the present study (Additional file 1: Table S1). The average sequencing coverage of these newly assembled plastomes ranged from 117 × to 2343 × , while plastome length ranged from 153,584 bp (*Chuniophoenix suoitienensis* A.J. Hend.) to 160,194 bp (*Borassus madagascariensis* (Jum. & H. Perrier) Jum. & H. Perrier). However, the species *Tahina spectabilis* J. Dransf. & Rakotoarinivo, the whole plastome, which was sequenced by Barrett et al. [47], has the smallest size (126,251 bp) among all the palm plastomes analyzed in the present study due to the loss of one copy of the inverted repeat. For the 49 accessions that have limited plastid sequences included in the incomplete-105 regions matrix (including 105 plastid regions from 349 accessions, among which 49 do not have complete or nearly complete plastome data), 381 sequences of plastid genes and intergenic regions were obtained in total from the National Center for Biotechnology Information (NCBI) database (Additional file 2: Table S2). The complete-coding (including 83 plastid coding regions of 300 accessions that have complete or nearly complete plastome data), complete-105 regions (including 105 plastid regions of 300 accessions that have complete or nearly complete plastome data), and incomplete-105 regions matrices had aligned lengths of 77,140 bp, 101,842 bp, and 101,931 bp, respectively, and with 1.34%, 1.28%, and 15.32% missing data, respectively.

### Phylogenetic relationships obtained from different data matrices

**Fig. 1:**
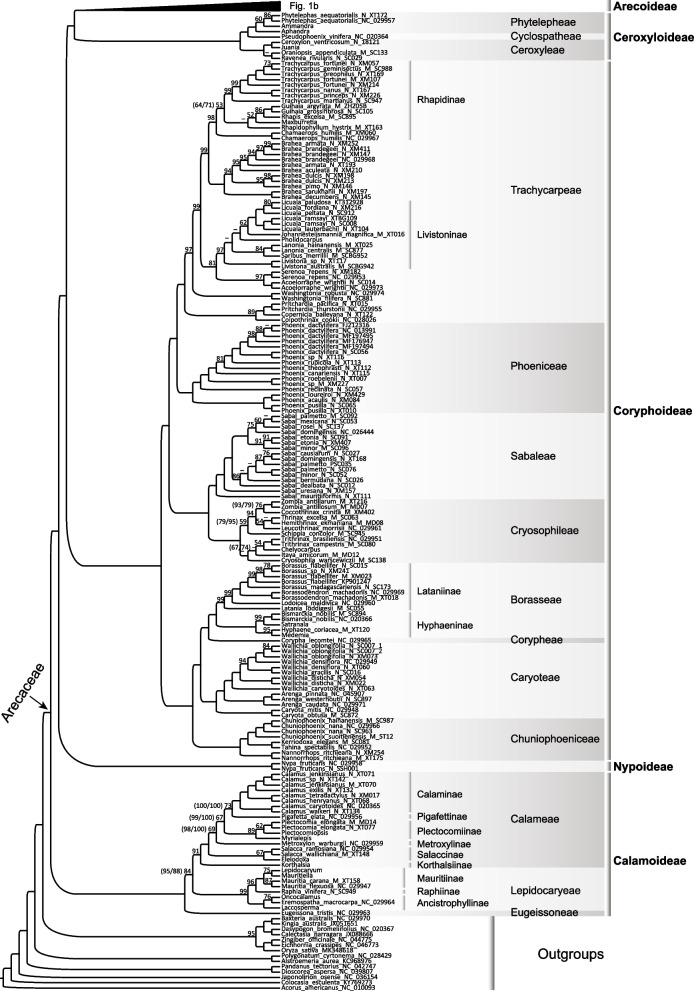

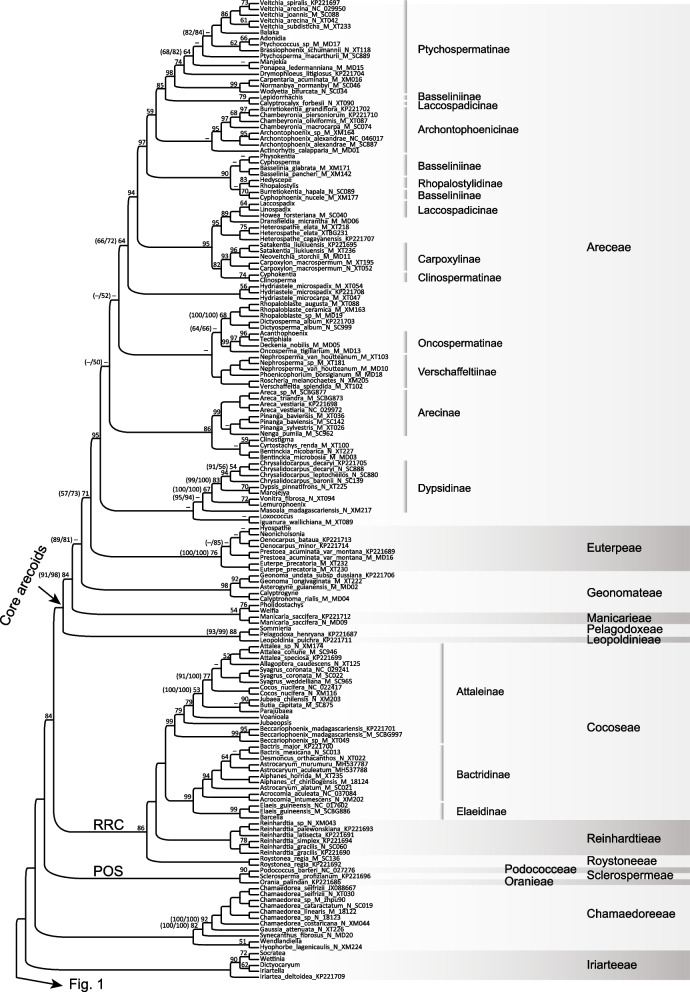
Maximum likelihood (ML) phylogenetic tree of Arecaceae inferred from the incomplete-105 regions matrix. Bootstrap support (BS) values inferior to 100% are shown, with dashes denoting a support inferior to 50%. Numbers in brackets indicate the BS values obtained from the analyses of the complete-coding/complete-105 matrices. Taxon names are followed by their GenBank accession numbers (sequences downloaded from NCBI) or voucher specimen information (plastomes newly obtained). For the 49 genera with a large proportion of missing data and for which individual plastid regions had to be retrieved from NCBI (see the “Methods” section), only the genus name is provided, but detailed sequence accession numbers can be found in Additional file 2: Table S2

Within Arecaceae, phylogenetic relationships among the five subfamilies and 28 tribes were consistent and mostly strongly supported (or highly supported defined here as Bootstrap (BS) ≥ 85%) in the trees obtained from all three matrices (Fig. 1; Additional files 4, 5: Figs. S1, S2), except several tribal-level nodes moderately (defined here as 70% ≤ BS < 85%) or weakly (defined here as BS < 70%) supported (Fig. 1; Additional files 4, 5: Fig. S1, S2), such as the sister relationships between the tribes Calameae and Lepidocaryeae (BS = 84%) within Calamoideae, between the RRC clade [66] and the core arecoids (BS = 84%), between (Areceae, Euterpeae) and the core core Geonomateae (BS < 50%), and between ((*Pholidostachys* H. Wendl. ex Hook. f., *Welfia* H. Wendl.), Manicarieae) and ((Areceae, Euterpeae), core Geonomateae) (BS = 84%) within Arecoideae in the incomplete-105 regions tree (Fig. 1), the crown of the RRC clade (BS = 82%) in the complete-coding tree (Additional file 4: Fig. S1), the sister relationship between (Areceae, Euterpeae) and the core core Geonomateae (BS = 81%) in the complete-105 regions tree (Additional file 5: Fig. S2), and the sister relationship between Areceae and Euterpeae (BSs = 71%, 57%, 73% in Fig. 1, Additional files 4, 5: Fig. S1, S2) in all three trees. Phylogenetic relationships at the subtribal level were also consistent and mostly strongly supported in all the trees (Fig. 1; Additional files 4, 5: S1, S2), except that several nodes within the tribe Calameae were weakly or moderately supported in the incomplete-105 regions tree (Fig. 1), and several nodes within Areceae were weakly or moderately supported in all the trees (Fig. 1b; Additional files 4, 5: Figs. S1, S2). The generic-level relationships within the family were also largely resolved with moderate to strong support (Fig. 1; Additional files 4, 5: Figs. S1, S2). Several generic-level nodes within the tribe Areceae showed conflicted topologies but were weakly supported in all the trees (Fig. 1b; Additional files 4, 5: Figs. S1, S2).

In the maximum likelihood (ML) tree based on the complete-105 regions matrix (Additional file 5: Fig. S2), stronger support values were recovered at most nodes compared with those from the complete-coding matrix (Additional file 4: Fig. S1). This was especially the case for the sister relationships between Mauritiinae and Raphiinae (from 80 to 99%; Additional files 4, 5: Figs. S1, S2), between ((Areceae, Euterpeae), Geonomateae) and Manicarieae (from 91 to 98%; Additional files 4, 5: Figs. S1, S2), and between Areceae and Euterpeae (from 57 to 73%; Additional files 4, 5: Figs. S1, S2). Tribal- and subtribal-level placements of the 49 genera with a large proportion of missing data were mostly resolved with strong support in the analysis based on the incomplete-105 regions matrix (Fig. 1). Among them, the stem nodes of 29 genera had bootstrap support values ≥ 70% (Fig. 1; Additional file 3: Table S3), including values ≥ 95% for *Acanthophoenix* H. Wendl., *Aphandra* Barfod, *Balaka* Becc., *Barcella* Trail ex Drude, *Calyptrogyne* H. Wendl., *Juania* Drude, *Laccosperma* Drude, *Medemia* Wurttemb. ex H. Wendl., *Satranala* J. Dransf. & Beentje, *Sommieria* Becc., and *Tectiphiala* H.E. Moore, which all have the proportion of missing data over 89% in the incomplete-105 regions matrix (Additional file 3: Table S3). However, some deeper nodes associated with the stem nodes of these 49 genera that have a large proportion of missing data obtained lower supports from the incomplete-105 regions matrix (Fig. 1) than from the complete-105 regions matrix (Additional file 5: Fig. S2), such as the sister relationships between the core Geonomateae and (Areceae, Euterpeae) (81% vs. < 50%), between subtribes Calaminae and Pigafettinae (100% vs. 73%), between Plectocomiinae and (Calaminae, Pigafettinae) (100% vs. 67%), and between Metroxylinae and ((Calaminae, Pigafettinae), Plectocomiinae) (100% vs. 69%).

### Phylogenetic results based on the incomplete-105 regions matrix

Considering the highly congruent topologies obtained across all the three ML analyses, we focus here on describing phylogenetic relationships mainly derived from the incomplete-105 regions matrix (Fig. 1), because this matrix included a more comprehensive taxon sampling compared with the other two matrices.

Arecaceae were placed within the commelinid monocots, and the monophyly of the family and the commelinid clade was both strongly supported (BS = 100%; Fig. 1). Within Arecaceae, the monophyly of all the five subfamilies was strongly supported (BSs = 100%), and phylogenetic relationships among these subfamilies were well-resolved with strong support values (BSs = 100%; Fig. 1). The subfamily Calamoideae was placed as sister to the remaining subfamilies and then followed successively by the monotypic subfamily Nypoideae and the second-largest subfamily Coryphoideae, which was placed as sister to the (Arecoideae, Ceroxyloideae) clade (Fig. 1).

Within Calamoideae, phylogenetic relationships among the three tribes (viz. Calameae, Eugeissoneae, and Lepidocaryeae) were well-resolved and the sister relationship between Calameae and Lepidocaryeae was moderately supported (BS = 84%, Fig. 1). Within the tribe Lepidocaryeae, the subtribe Ancistrophyllinae was placed as sister to the (Mauritiinae, Raphiinae) clade, and relationships among the three subtribes were strongly supported (BSs ≥ 96%; Fig. 1). Within Calameae, the subtribe Korthalsiinae was placed as sister to the remaining members of the tribe with high support (BS = 91%; Fig. 1), and then followed by the subtribe Salaccinae (BS = 100%; Fig. 1), and Metroxylinae was placed as sister to the ((Calaminae, Pigafettinae), Plectocomiinae) clade. Relationships among the latter four subtribes were weakly or moderately supported.

Two major clades were recovered in the subfamily Coryphoideae: ((Phoeniceae, Trachycarpeae), (Cryosophileae, Sabaleae)) and (((Borasseae, Corypheae), Caryoteae), Chuniophoeniceae), with relationships at the tribal level all strongly supported (BSs = 100%; Fig. 1). The monophyly of each of these tribes was also strongly supported (BSs = 100%), except for Corypheae, represented here by only one accession of *Corypha lecomtei* Becc. ex Lecomte. Within Trachycarpeae, the phylogenetic positions of the seven genera (viz. *Acoelorrhaphe* H. Wendl., *Serenoa* Hook.f., *Brahea* Mart., *Colpothrinax* Schaedtler, *Copernicia* Mart. ex Endl., *Pritchardia* Seem. & H. Wendl. ex H. Wendl., and *Washingtonia* H. Wendl.) that were not classified at subtribal level [40] were well-resolved with strong support. The clade ((*Copernicia*, *Pritchardia*), *Colpothrinax*) (BS = 89%; Fig. 1) was placed as sister to all the other members of Trachycarpeae with strong support (BS = 100%), then followed by the genus *Washingtonia*, also with strong support (BS = 97%). In addition, within this tribe, the genus *Brahea* was sister to the subtribe Rhapidinae with strong support (BS = 99%), and the (*Acoelorraphe*, *Serenoa*) subclade (BS = 97%) was sister to the subtribe Livistoninae (BS = 81%). Monophyly of the two subtribes Hyphaeninae and Lataniinae, in the tribe Borasseae, was strongly supported (BSs ≥ 99%).

Within the subfamily Ceroxyloideae, relationships among all the three tribes were well-resolved with strong support (BSs = 100%; Fig. 1), with the tribe Ceroxyleae placed as sister to (Cyclospatheae, Phytelepheae). Furthermore, relationships among all eight genera of the subfamily were most strongly supported except for two nodes: (*Ammandra* O.F. Cook, *Phytelephas* Ruiz & Pav.) (BS = 60%) and (*Oraniopsis* (Becc.) J. Dransf., A.K. Irvine & N.W. Uhl, *Ravenea* C.D. Bouché) (BS < 50%).

In the largest subfamily Arecoideae (Fig. 1b), the tribe Iriarteeae was placed as sister to the rest of the subfamily, followed successively by Chamaedoreeae and ((Podococceae, Sclerospermeae), Oranieae), i.e., the POS clade [66], and these relationships were all strongly supported (BSs = 100%). Relationships among the three tribes within the POS clade were also well resolved with strong support (BSs ≥ 90%). The remaining members of Arecoideae formed two major clades: the ((Cocoseae, Reinhardtieae), Roystoneeae) (RRC [66]) clade and the core arecoids, the monophyly of which was strongly supported (BS = 86% and 100%, respectively). Within the RRC clade, the sister relationship between Cocoseae and Reinhardtieae, and the relationships among the three subtribes ((Bactridinae, Elaeidinae), Attaleinae) of Cocoseae, all obtained strong support (BSs ≥ 99%). Within the core arecoids, the two tribes Leopoldinieae and Pelagodoxeae formed a well-supported clade (BS = 88%) and were placed as sister to the rest of the core arecoids with high support (BS = 100%). The tribe Manicarieae formed a weakly supported clade (BS = 54%) with two genera (*Pholidostachys* and *Welfia*) of Geonomateae, while the core Geonomateae was sister to (Areceae, Euterpeae) with no support (BS < 50%). Additionally, the sister relationship between Areceae and Euterpeae was moderately supported (BS = 71%).

Within the largest tribe Areceae, the monophyly of some subtribes, such as Arecinae (BS = 99%), Carpoxylinae (93%), Oncospermatinae (99%), Ptychospermatinae (98%), and Verschaffeltiinae (100%) received strong support. Some lineages also received strong support, such as ((*Calyptrocalyx* Blume, *Lepidorrhachis* O.F. Cook), Ptychospermatinae) (BS = 85%), (Basseliniinae (except *Lepidorrhachis*), Rhopalostylidinae) (BS = 90%), (((Laccospadicinae (except *Calyptrocalyx*), *Dransfieldia* W.J. Baker & Zona), *Heterospathe* Scheff.), (Carpoxylinae, Clinospermatinae)) (BS = 95%), and (((*Clinostigma* H. Wendl., *Cyrtostachys* Blume), *Bentinckia* Berry ex Roxb.), Arecinae) (BS = 86%). However, the monophyly of both Basseliniinae and Laccospadicinae was not supported here, because the genera *Calyptrocalyx* of Laccospadicinae and *Lepidorrhachis* of Basseliniinae formed a moderately supported clade (BS = 79%) that was sister to Ptychospermatinae with high support (BS = 85%), and was distantly related to the other members of either Basseliniinae or Laccospadicinae. Additionally, the monophyletic subtribe Rhopalostylidinae (BS = 83%) was nested deeply within a clade composed of the members of Basseliniinae except *Lepidorrhachis*, although the placement of Rhopalostylidinae within this clade was weakly supported. Among the ten genera that were not classified at the subtribal level, the two genera *Dransfieldia* and *Heterospathe* formed a highly supported clade (BS = 95%), which was sister to (Carpoxylinae, Clinospermatinae) with high support (BS = 95%). The genus *Hydriastele* H. Wendl. & Drude represents an independent lineage within Areceae and was weakly supported as the sister of a large clade comprised by Archontophoenicinae, Basseliniinae, Carpoxylinae, Clinospermatinae, *Dransfieldia*, *Heterospathe*, Laccospadicinae, Rhopalostylidinae, Ptychospermatinae (BS = 64%). The two genera *Dictyosperma* H. Wendl. & Drude and *Rhopaloblaste* Scheff. formed a weakly supported clade (BS = 69%) that was sister to Oncospermatinae (BS < 50%). The three genera *Bentinckia*, *Clinostigma*, and *Cyrtostachys* formed a clade, but with no support (BS < 50%), that was sister to Arecinae with high support (BS = 86%). Finally, the genus *Iguanura* Blume was recovered as sister to *Loxococcus* H. Wendl. & Drude, and then they were collectively sister to the subtribe Dypsidinae, but relevant nodes obtained no support (BS < 50%).

## Discussion

### Insights into the plastid phylogenomic resolution of the palms based on a nearly complete generic-level sampling

Our study represents the first phylogenomic analysis of palms based on both a comprehensive generic sampling and a large number of plastid genes. In the present study, several nodes weakly or moderately supported in the analysis of the incomplete-105 regions matrix obtained strong support in analyses of the other two matrices, such as the sister relationships between the tribes Calameae and Lepidocaryeae within Calamoideae (BS = 84% in the incomplete-105 regions tree, Fig. 1; BSs = 95%, 88%, in the complete-coding tree and complete-105 regions tree, Additional files 4, 5: Figs. S1, S2), and between (Areceae, Euterpeae) and the core Geonomateae within Arecoideae (BS < 50% in the incomplete-105 regions tree, Fig. 1; BS = 89% in the complete-coding tree, Additional file 4: Fig. S1). Our results are largely congruent with previous plastid phylogenetic analyses [47, 48, 57, 58, 64] but provide higher support for some tribal and generic relationships, especially those among Geonomateae, Leopoldinieae, Manicarieae, and Pelagodoxeae (Fig. 1; Additional files 4, 5: Figs. S1, S2).

The relationships among all the palm subfamilies and tribes were resolved with high support in the present study, except the sister relationship between Areceae and Euterpeae, which obtained moderate support (Fig. 1; Additional file 5: Fig. S2). However, this sister relationship was strongly supported in previous studies based on both nuclear genomic data [39] and plastome data [48, 58], as well as in a recent study based on a combined analysis of three nuclear regions (*RPB2*, *CISP4*, *WRKY6*) and one plastid region (*trnT-trnD*) [75]. Relationships among subtribes and genera of the family were also mostly resolved with moderate or high support (Fig. 1; Additional files 4, 5: Figs. S1, S2). However, clarifying some subtribal and generic relationships within the largest tribe Areceae is still a great challenge. Subtribes and genera within this tribe may have undergone rapid radiations, and perhaps including more species combined with the inclusion of more non-coding sequences (non-CDS) into further plastid phylogenomic analyses focused on this tribe would help improve the resolution of nodes that remain unclear, for instance, the placements of the genera *Actinorhytis*, *Iguanura*, *Loxococcus*, *Ponapea* and *Ptychosperma*, and subtribes Oncospermatinae, Verschaffeltiinae.

Previous theoretical [19, 76] and empirical studies [13, 14] have demonstrated that increased taxon sampling can improve resolution and support in phylogenetic analyses, even if including taxa with relatively slow substitution rates and large proportion of missing data. This was further supported by the present study. Our results from the analysis of the incomplete-105 regions matrix showed that moderate or strong support was obtained for the phylogenetic placements of nearly 60% of the 49 genera that have a large proportion of missing data (approximately up to 90% or even higher; Fig. 1; Additional file  3: Table S3). The well-supported phylogenetic positions of these genera indicate that their limited sequence data may be enough to clarify their placements on the phylogenetic tree. This is supported by the fact that, for many of these genera, the same phylogenetic position was recovered with high support in the KTLE nuclear tree (PP = 1.00) [60]. This was notably the case for *Acanthophoenix*, *Balaka*, *Barcella*, *Calyptrogyne*, *Juania*, *Korthalsia* Blume, *Medemia*, *Myrialepis* Becc., *Satranala*, *Sommieria*, and *Tectiphiala*. Thus, the scaffold approach [14] of adding taxa with a large proportion of missing data into the analysis seems to be a good method to overcome the drawback of incomplete taxon sampling in phylogenomic studies.

### Comparison between plastid and nuclear phylogenomic trees

The current version of the KTLE tree [60] has not yet been used as the basis for an in-depth discussion of palm relationships. Nevertheless, it provides important insight into the generic-level relationships of palms and is largely congruent with other published nuclear trees, such as the generic-level relationships within the subfamily Calamoideae [49] and the tribe Geonomateae [63], the tribal-level relationships within Calamoideae [49] and Ceroxyloideae [66], and the relationships among major clades of the subfamily Arecoideae [39] and among the five palm subfamilies [39, 49]. The plastome tree inferred here is complementary to these nuclear trees and brings some advantages because it is less likely to be biased by very high rates of substitution, paralogy, or incomplete lineage sorting, which can result in phylogenetic inference errors or conflicts among gene trees. On the other hand, comparing plastome and nuclear trees can reveal traces of past hybridization events that have disappeared from nuclear genomes, such as those involved in chloroplast capture [21, 77]. Considering these nuclear and plastid trees together thus can strengthen our understanding of the palm phylogeny and evolutionary history.

Phylogenetic topologies among all the five subfamilies, among major clades within Arecoideae, among most tribes within Coryphoideae, and among most subtribes and genera in the family were consistent in analyses based on both plastid (Fig. 1) [47, 48, 58] and nuclear data [39, 49, 59, 60, 63, 66, 78] and also mostly obtained strong supported. Additionally, although the sister relationships between Phoeniceae and Trachycarpeae within Coryphoideae recovered in the plastome tree (Fig. 1) were not derived from the analysis of 353 nuclear genes [59, 60], but it was highly supported in Faurby et al.’s [25] supertree analysis as well as Cano et al.’s [79] study based on analysis of four nuclear regions (*CISP4*, *CISP5*, *PRK*, *RPB2*) and one plastid region (*matK*).

**Fig. 2:**
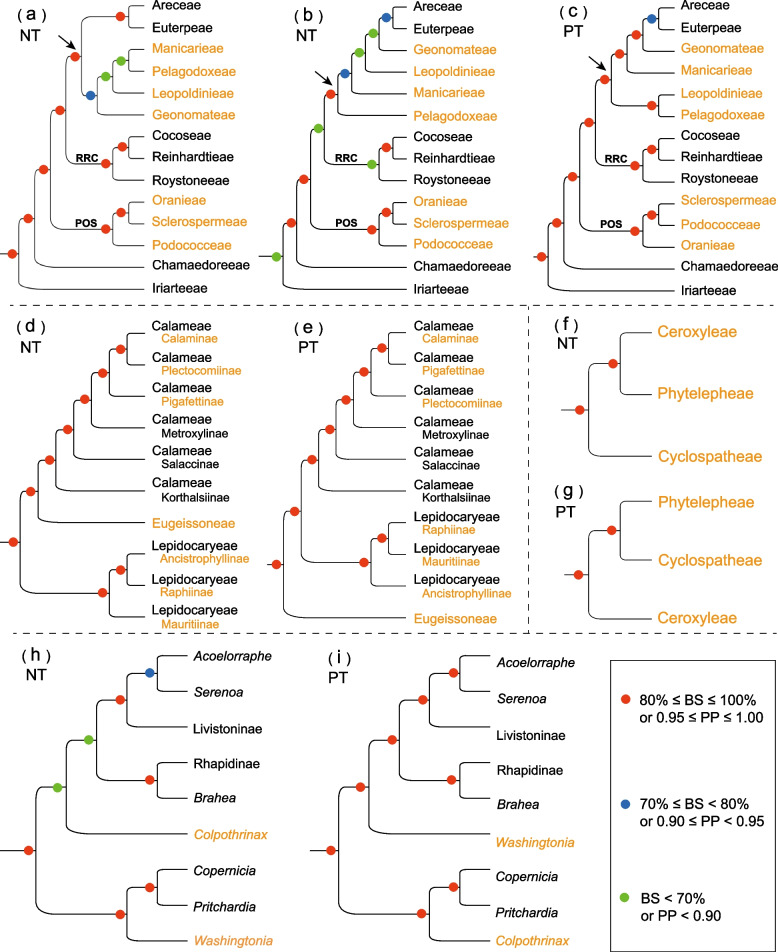
Comparison between the palm topologies derived from nuclear and plastome data. **a**‒**c** Tribal relationships in Arecoideae, the arrowhead indicates the crown of the core arecoids; **d**,** e** tribal and subtribal relationships in Calamoideae; **f**, **g** tribal relationships in Ceroxyloideae; **h**,** i** subtribal relationships in Trachycarpeae (Coryphoideae); NT nuclear topology, PT plastome topology; NT were obtained **a** from the maximum likelihood analysis of Comer et al. [39]; **b**, **h** from the multispecies coalescent analysis of KTLE [60] (KTLE tree); **d** from the multispecies coalescent analysis of Kuhnhäuser et al. [49]; and **f** from the maximum parsimony analysis of Baker et al. [66]; PT of **c**, **e**, **g**, and **i** were all obtained from the present study

Widespread nuclear-plastid discordance within each palm subfamily (excluding the monotypic subfamily Nypoideae) was observed at different taxonomic levels between our plastome tree (Fig. 1; Additional files 4, 5: Figs. S1, S2) and previously published nuclear trees [39, 49, 59, 60, 63, 66]. Detailed information about the discordances regarding relationships among tribes and subtribes is summarized in Fig. 2. For some of these discordances, the nuclear topology was most often better supported by morphological evidence than the plastid topology. For example, Calaminae and Plectocomiinae, which were placed as sisters in the nuclear trees [49, 59, 60] but not in our plastid trees (Fig. 2d), are mostly climbing palms that have bi-symmetric and aperturate pollen grains, while members of Pigafettinae are massive trees with sub-actinomorphic and inaperturate pollen grains [43]. However, for other discordances, the plastid topology is better supported by morphological evidence than the nuclear topology. This is the case for Calameae and Lepidocaryeae, which are sisters in our plastid topology (Fig. 1; Fig. 2e) but not in previous nuclear topologies (Fig. 2d; [49, 59, 60]). These tribes are characterized by flowers not borne in a cupule of bracts, less than 20 stamens and fruits lacking an endocarp, while the tribe Eugeissoneae is characterized by its large flowers that are born in a cupule of bracts, more than 20 stamens and fruits with a thick endocarp [43]. Finally, it is interesting to note that the plastid topology ((Podococceae, Sclerospermeae), Oranieae) is well supported by the geographic distribution of the tribes, as Podococceae and Sclerospermeae are endemic to western Africa, while Oranieae are mainly distributed from southeast Asia to New Guinea, with only three species in Madagascar [43].

Nuclear-plastid discordance has been reported widely in other angiosperm lineages both at deep and shallow nodes, such as the asterids [20], Caryophyllales [13, 80], Fagales [21], Fabaceae [31, 32], Orchidaceae [35], and *Magnolia* L. (Magnoliaceae) [81], and the discordance is mostly interpreted as resulting from hybridization and/or ILS [20, 21, 77, 81]. In the case of palms, our study suggests that the conflicting nuclear and plastid topologies could be mainly due to incomplete lineage sorting during rapid radiations, because the nodes involved tend to be associated with very short internal branches and long external branches (Additional file 6: Fig. S3). This is notably the case for the diversifications among major lineages within the core arecoids and the POS clade in Arecoideae, among tribes in Calamoideae, and along the backbone of Trachycarpeae in Coryphoideae. Incomplete lineage sorting is expected to make ancestral genetic polymorphisms persist during evolutionary radiations and could therefore have induced the observed phylogenetic incongruences [82]. However, such patterns of short internal branch coupled with long external branches resulting from fast diversification combined with a lack of species-level sampling can make phylogenetic inference prone to errors, and incongruences around these nodes may thus also be due to tree estimation errors. Comprehensive species-level sampling will be instrumental to avoid this pitfall in further studies. On the other hand, hybridization is another phenomenon that can lead to phylogenetic discordance, and it has been shown that palms can hybridize at least in gardens and have done so in the past [83]. Accordingly, some palm lineages involved in nuclear-plastid discordance have overlapping or closely adjacent distribution ranges, which may facilitate hybridization between them. This is for instance the case of Geonomateae, Leopoldinieae, and Manicarieae (core arecoids), whose members often co-occur in Amazonia [43]. Hybridization therefore seems another plausible explanation for the palm nuclear-plastid discordances observed in the present study. Moreover, some of the conflicted topologies are highly supported in both the plastome tree (Fig. 1) and nuclear trees [39, 49, 59, 60, 66], suggesting possible hybridization events involved in chloroplast capture may have occurred in relevant nodes, such as those observed among tribes within the two subfamilies Calamoideae (Fig. 2d, e) and Ceroxyloideae (Fig. 2f, g), and among the three tribes within the POS clade (Fig. 2a‒c). Further phylogenetic studies and gene tree frequency analyses will be necessary to enable the accurate detection and quantification of ILS and hybridization across the palms and to achieve higher phylogenetic resolution.

### Implications for palm taxonomy

The palm family has benefited from considerable systematic studies over recent decades [24, 39–43, 48–50, 55–57, 61]. This rich history has led to continual improvements in our understanding of palm evolution and relationships, which underpins the current taxonomic classification. As circumscribed in the latest taxonomic treatment of the palms provided by Baker and Dransfield [40], members of the family are classified into five subfamilies and 28 tribes, and most genera within large tribes are also classified at the subtribal level.

Monophyly of all the five subfamilies and tribes circumscribed in Baker and Dransfield [40] was mostly highly supported here by plastome data. An exception is the tribe Geonomateae in the subfamily Arecoideae (Fig. 1), for which the two genera *Pholidostachys* and *Welfia* did not fall within the core Geonomateae but were sister to the tribe Manicarieae with weak support (BS = 54%; Fig. 1). The sister relationship between (*Pholidostachys*, *Welfia*) and Manicarieae was also reported previously in an analysis based on four plastid regions and extensive generic sampling, with higher support (BS = 89%) [57]. However, the monophyly of Geonomateae (including *Welfia* and *Pholidostachys*) was highly supported in nuclear phylogenomic studies [60, 63], with *Welfia* and *Pholidostachys* successively sister to the remaining members of Geonomateae with weak support in the KTLE tree [60] and high support in an analysis based on 795 nuclear genes [63].

The taxonomic circumscriptions of subtribes recognized within some large palm tribes by Baker and Dransfield [40] were also supported in the present plastome study (Fig. 1), with the exception of subtribes Basseliniinae and Laccospadicinae in the largest tribe Areceae. Similar results were also recovered in the KTLE nuclear tree [60]. Our results, combined with those from the KTLE topology [60] and other recent phylogenetic studies of the palms [25, 64, 66, 79], indicate the need for some updates to the taxonomic classification of the family.

1. The highly supported placement of the genus *Calyptrocalyx* distant from the remaining members of the subtribe Laccospadicinae recovered in the plastome analyses (Fig. 1b) was also supported in the analysis of Baker et al. [66] based on two nuclear regions (*PRK*, *RPB2*) and the KTLE nuclear tree [60]. Thus, the circumscription of Laccospadicinae should be revised and the subtribal placement of *Calyptrocalyx* should be reassessed.
2. The monophyletic subtribe Rhopalostylidinae was nested deeply within the Basseliniinae with high support as recovered in the present plastome tree (Fig. 1b). A similar result for the placement of Rhopalostylidinae within Basseliniinae was also highly supported in Faurby et al. [25], and the placement of the genus *Rhopalostylis* (Rhopalostylidinae) within Basseliniinae was also highly supported in the KTLE nuclear tree [60]. We recommend that the Basseliniinae should be expanded to include Rhopalostylidinae.
3. The phylogenetic positions of multiple palm genera which had not been classified to subtribes before have been clarified in our study with moderate to strong support (BSs ≥ 70%; Fig. 1; Additional files 4, 5: Figs. S1, S2). These include *Bentinckia*, *Cyrtostachys*, *Dictyosperma*, *Dransfieldia*, *Heterospathe*, *Hydriastele*, and *Rhopaloblaste* in the tribe Areceae and *Acoelorraphe*, *Brahea*, *Colpothrinax*, *Copernicia*, *Pritchardia*, *Serenoa*, and *Washingtonia* in the tribe Trachycarpeae. The isolated phylogenetic positions of these genera out of all currently well-defined palm subtribes were also recovered in Faurby et al. [25] and in the KTLE nuclear tree [60], and similar results for the placements of some of these genera were also supported in recent studies focused on different palm lineages [25, 64, 66, 79]. Improvements to the subtribal classification of palms may be achievable in light of these new results, especially in concert with the growing body of nuclear phylogenomic evidence.

## Conclusions

Our study is the most comprehensive plastome-based phylogenomic analysis yet conducted for palms. Our results improve upon previous plastid-based studies that were limited either in taxon or in locus sampling, providing resolution and support throughout the palm family, with most tribe- and genus-level nodes strongly supported. The plastome data provided here complement previously reported nuclear trees and our understanding of the evolutionary history of the palms, thus strengthening our understanding of palm relationships. The robust phylogenetic hypothesis of the palms reconstructed here will support future studies on the biogeography, classification, diversification, and evolution of the family. The present phylogenomic investigation of the palms provides a case study of how to use phylogenomics to disentangle relationships among lineages of a large angiosperm family. Comprehensive, species-level, nuclear phylogenomic studies of palms are also nearing completion [84] and will provide important opportunities for the evaluation of nuclear-plastid discordance.

## Methods

### Taxon sampling

Plastomes of 210 accessions representing 182 species and 111 genera of Arecaceae were newly sequenced for this project, and detailed information about these accessions and voucher specimens is provided in Additional file 1 (Table S1). We further added complete or nearly complete plastid genome sequences from NCBI (https://www.ncbi.nlm.nih.gov/), corresponding to 76 accessions representing 71 species and 63 genera of the palm family (45 of these genera were duplicate with those newly sequenced here) (Additional file 1: Table S1). Most of these were derived from the phylogenomic studies conducted by Barrett et al. [47] and Comer et al. [48]. In addition, another 49 palm accessions representing 49 genera that have at least five plastid DNA regions in NCBI (www.ncbi.nlm.nih.gov) were also included (Additional file 2: Table S2), most of which were from Baker et al. [24]. In total, the final taxon sampling included 335 palm accessions representing 276 species and 178 genera, accounting for 98.3% of all currently circumscribed palm genera, and representing all the subtribes, tribes, and subfamilies [40]. Three palm genera, viz. *Jailoloa* Heatubun & W.J. Baker, *Sabinaria* R. Bernal & Galeano, and *Wallaceodoxa* Heatubun & W.J. Baker, which are recently described genera [40], were not sampled here because of the lack of DNA material or published plastid sequence data, but their phylogenetic positions within the family were resolved previously in analyses based mainly on nuclear data [60, 79, 85, 86]. Additionally, complete plastome sequences of four genera of the family Dasypogonaceae and ten genera representing the other ten monocot orders (Additional file 1: Table S1) were selected as outgroups based on the phylogenetic framework provided by Givnish et al. [87] and Li et al. [52].

### Plastome DNA extraction, sequencing, assembly, and annotation

Total genomic DNAs were extracted from silica-dried leaves following the CTAB protocol of Doyle and Doyle [88]. DNAs were sheared to approximately 500-bp fragments through ultrasonic treatment and used to construct short-insert libraries following the manufacturer’s protocol (NEBNext® Ultra II™DNA Library Prep Kit for Illumina®) and sequenced from both ends on the Illumina HiSeq 2500 platform at Beijing Genomics Institute (BGI, Shenzhen, China) to generate 2 × 150-bp sequencing reads. Approximately 3 GB of raw data was generated for each sample. Plastid reads were assembled using the software GetOrganelle [89] with parameter settings as follows: “-t 30 -R 15 -k 75, 85, 95, 105 -F embplant_pt,” using the plastid genomes of *Nypa fruticans* Wurmb (GenBank accession number: NC_029958) and *Veitchia arecina* Becc. (NC_029950) as references. All the plastid genes were then annotated using the software PGA [90], with the annotated plastome of *Amborella trichopoda* Baill. (NC_005086) as a reference, following the recommendation of Qu et al. [90]. GenBank accession numbers of the complete or nearly complete plastome newly sequenced here as well as those obtained from NCBI are listed in Additional file 1 (Table S1), and the GenBank accession numbers corresponding to the sequences from the 49 accessions with few plastid DNA regions available are listed in Additional file 2 (Table S2).

### Phylogenetic dataset construction

Coding regions of 79 protein-coding genes, four ribosomal RNA genes, six transfer RNA genes with sequence lengths above 200 bp (viz. *trnA-UGC*, *trnG-UCU*, *trnI-GAU*, *trnK-UUU*, *trnL-UAA*, and *trnV-UAC*), and intron regions of all 12 intron-containing coding genes (viz. *atpF*, *clpP*, *ndhA*, *ndhB*, *petB*, *petD*, *rpl12*, *rpl16*, *rpoC1*, *rps12*, *rps16*, *ycf3*) were extracted from the plastomes as conducted in Geneious 11.1.5 [91]. Additionally, to coordinate with the 49 accessions for which at least five plastid regions were available, sequences of four intergenic spacer regions (viz. *rps15-ycf1*, *trnD-trnT*, *trnL-F*, and *trnQ-rps16*) were also extracted. Sequence matrices corresponding to each region were aligned independently using the plugin of MAFFT [92] in Geneious with the default settings. The removal of ambiguously aligned sites in 17 matrices (including that of the *trnK-UUU* region, all the 12 intron-containing and four intergenic regions) was conducted in Gblocks 0.91b [93], with the option “Allowed Gap Positions” set as “All.”

Three different data sets were constructed: (1) a complete-coding matrix, including all 83 coding regions (viz. 79 protein-coding genes and four ribosomal RNA genes) of 300 accessions (including 129 palm genera and 28 palm tribes) that possessed complete or nearly complete plastome data; (2) a complete-105 regions matrix (including 105 plastid regions of 300 accessions), constructed by adding the complete-coding matrix to the other 22 plastid regions (i.e., six rRNA regions, 12 intron-containing regions, and four intergenic spacer regions); and (3) an incomplete-105 regions matrix (including 105 plastid regions of 349 accessions, among which 49 accessions had limited plastome data), constructed based on the complete-105 regions matrix, with the additional inclusion of the 49 accessions (representing 49 genera) with at least five plastid regions available in NCBI. Information about the proportion of missing data for each of the 49 genera included in the incomplete-105 regions matrix is presented in Additional file 3 (Table S3).

### Phylogenetic analyses

The plastome has long been considered to comprise a single linkage group [94, 95], and thus, plastid genes are usually concatenated in order to maximize the overall phylogenetic signal [5, 13, 34]. In the present study, all three matrices were analyzed under an unpartitioned scheme. We inferred phylogenetic trees from the three matrices, using the maximum likelihood (ML) approach implemented in RAxML-HPC2 (8.1.24) [96] on the CIPRES cluster [97], employing the GTR + Γ model with the default number of rate categories (C = 25). We conducted a rapid bootstrap (BS) analysis with 1000 pseudoreplicates. The trees obtained were visualized and edited using FigTree v.1.4.4 [98]. The alignments and ML trees are available from figshare [99].

## Supplementary Information

**Additional file 1: Table S1.** List of accessions sampled, with voucher specimens, GenBank accession numbers and the length of plastomes newly sequenced. Sequences marked with ‘✱’ were obtained from NCBI (https://www.ncbi.nlm.nih.gov/).

**Additional file 2:  Table S2.** Accessions of the 49 genera with few plastid DNA regions available and therefore a large proportion of missing data in the incomplete-105 regions matrix, with GenBank accession numbers of sequences used in the present study. The character ‘—’ indicates that the sequence was unavailable from NCBI (https://www.ncbi.nlm.nih.gov/) and treated as missing data in the matrix.

**Additional file 3: Table S3.** Proportion of missing data for the 49 accessions (genera) with few DNA sequences available, compared to the total length of the incomplete-105 regions matrix, and bootstrap value for the stem nodes of these accessions (genera) obtained from maximum likelihood (ML) analysis of the incomplete-105 regions matrix (Fig. 1).

**Additional file 4: Fig. S1.** Maximum likelihood phylogenetic tree of Arecaceae inferred from the complete-coding matrix. Bootstrap values inferior to 100% are shown, with dashes denoting a support inferior to 50%.

**Additional file 5: Fig. S2.** Maximum likelihood phylogenetic tree of Arecaceae inferred from the complete-105 regions matrix. Bootstrap values inferior to 100% are shown, with dashes denoting a support inferior to 50%.

**Additional file 6: Fig. S3.** Maximum likelihood phylogenetic tree (including branch lengths) of Arecaceae inferred from the incomplete-105 regions matrix.

## Data Availability

All data generated in this study are available on the NCBI. The GenBank accession numbers are listed in Additional file 1: Table S1. All plant material collected for this study came from plants grown in a botanical garden. Sequence alignments underlying analyses and phylogenetic trees are available from figshare (https://doi.org/10.6084/m9.figshare.20489916) [99].
