# Supplementary material for: A plastid phylogenomic framework for the palm family (Arecaceae)
Source: BMC Biol. 2023 Mar 8;21:50. doi: 10.1186/s12915-023-01544-y (PMC9993706; doi:10.1186/s12915-023-01544-y)
Supplement: Supplementary file 1 — Additional file 1: Table S1. List of accessions sampled, with voucher specimens, GenBank accession numbers and the length of plastomes newly sequenced. Sequences marked with ‘✱’ were obtained from NCBI (https://www.ncbi.nlm.nih.gov/). [file 12915_2023_1544_MOESM1_ESM.docx]

**Table S1** List of accessions sampled, with voucher specimens, GenBank accession numbers and the length of plastomes newly sequenced. Sequences marked with ‘✱’ were obtained from NCBI (<https://www.ncbi.nlm.nih.gov/>).

| **Species** | **Voucher specimen** | **GenBank accession number** | **The length of plastome newly sequenced (bp)** |
| --- | --- | --- | --- |
| *Acoelorraphe wrightii* (Griseb. & H. Wendl.) H. Wendl. ex Becc. | N_SC014 (IBSC) | ON248721 | 158,609 |
| *Acrocomia intumescens* Drude | N_XM202 (IBSC) | ON248616 | 156,590 |
| *Actinorhytis calapparia* (Blume) H. Wendl. & Drude ex Scheff. | M_MD01 (IBSC) | ON248722 | 158,205 |
| *Aiphanes* cf *chiribogensis* Borchs. & Balslev | M_18124 (IBSC) | ON248723 | 155,486 |
| *Aiphanes horrida* (Jacq.) Burret | M_XT235 (IBSC) | ON248724 | 155,707 |
| *Allagoptera caudescens* (Mart.) Kuntze | N_XT125 (IBSC) | ON248725 | 155,146 |
| *Archontophoenix sp* | M_XM164 (IBSC) | OM743238 | 155,199 |
| *Archontophoenix alexandrae* (F. Muell.) H. Wendl. & Drude | M_SC887 (IBSC) | ON248726 | 159,077 |
| *Areca sp* | M_SCBG877 (IBSC) | ON248776 | 156,136 |
| *Areca triandra* Roxb. ex Buch.-Ham. | M_SCBG873 (IBSC) | ON248727 | 157,447 |
| *Arenga westerhoutii* Griff. | N_SC897 (IBSC) | ON248617 | 159,872 |
| *Asterogyne guianensis* Granv. & A.J. Hend. | M_MD02 (IBSC) | ON248728 | 158,819 |
| *Astrocaryum alatum* H.F. Loomis | M_SC021 (IBSC) | OM743239 | 155,797 |
| *Attalea cohune* Mart. | M_SC946 (IBSC) | ON248729 | 154,486 |
| *Attalea sp* | N_XM174 (IBSC) | ON248680 | 155,354 |
| *Bactris mexicana Mart.* | N_SC013 (IBSC) | ON248618 | 156,774 |
| *Basselinia glabrata* Becc. | M_XM171 (IBSC) | ON248730 | 159,027 |
| *Basselinia pancheri* (Brongn. & Gris) Vieill. | M_XM142 (IBSC) | ON248731 | 158,964 |
| *Beccariophoenix madagascariensis* Jum. & H. Perrier | M_SCBG997 (IBSC) | ON248732 | 154,383 |
| *Beccariophoenix sp* | M_XT049 (IBSC) | ON248733 | 154,595 |
| *Bentinckia nicobarica* (Kurz) Becc. | N_XT227 (IBSC) | ON248619 | 158,740 |
| *Bentinckia nicobarica* (Kurz) Becc. | M_MD03 (IBSC) | ON248734 | 158,708 |
| *Bismarckia nobilis* Hildebrandt & H. Wendl. | M_SC894 (IBSC) | ON248620 | 158,171 |
| *Borassodendron machadonis* (Ridl.) Becc. | M_XT018 (IBSC) | ON248621 | 158,092 |
| *Borassus flabellifer* L. | N_SC015 (IBSC) | ON248622 | 160,018 |
| *Borassus flabellifer* L. | M_XM023 (IBSC) | ON248735 | 160,017 |
| *Borassus madagascariensis* (Jum. & H. Perrier) Jum. & H. Perrier | N_SC173 (IBSC) | ON248623 | 160,194 |
| *Borassus sp* | N_XM241 (IBSC) | ON248624 | 160,012 |
| *Brahea aculeata* (Brandegee) H.E. Moore | N_XM210 (IBSC) | ON248736 | 158,913 |
| *Brahea armata* S. Watson | N_XM252 (IBSC) | ON248625 | 158,842 |
| *Brahea armata* S. Watson | N_XT193 (IBSC) | ON248626 | 158,763 |
| *Brahea brandegeei* (Purpus) H.E. Moore | N_XM147 (IBSC) | ON248627 | 158,857 |
| *Brahea brandegeei* (Purpus) H.E. Moore | N_XM411 (IBSC) | ON248628 | 158,818 |
| *Brahea decumbens* Rzed. | N_XM145 (IBSC) | ON248629 | 158,767 |
| *Brahea dulcis* (Kunth) Mart. | N_XM198 (IBSC) | ON248630 | 158,650 |
| *Brahea dulcis* (Kunth) Mart. | N_XM213 (IBSC) | ON248631 | 158,612 |
| *Brahea pimo* Becc. | N_XM146 (IBSC) | ON248632 | 158,515 |
| *Brahea sarukhanii* H.J. Quero | N_XM197 (IBSC) | ON248633 | 158,795 |
| *Brassiophoenix schumannii* (Becc.) Essig | N_XT118 (IBSC) | ON248634 | 158,975 |
| *Burretiokentia hapala* H.E. Moore | N_SC089 (IBSC) | ON248737 | 158,861 |
| *Butia capitata* (Mart.) Becc. | M_SC875 (IBSC) | ON248738 | 154,420 |
| *Calamus exilis* Griff. | N_XT132 (IBSC) | ON248635 | 158,225 |
| *Calamus henryanus* Becc. | N_XT068 (IBSC) | ON248636 | 157,536 |
| *Calamus jenkinsianus* Griff. | N_XT071 (IBSC) | ON248648 | 158,209 |
| *Calamus jenkinsianus* Griff. | M_XT070 (IBSC) | ON248739 | 157,261 |
| *Calamus sp* | N_XT142 (IBSC) | ON248649 | 158,233 |
| *Calamus tetradactylus* Hance | N_XM017 (IBSC) | ON248740 | 157,883 |
| *Calamus walkeri* Hance | N_XT134 (IBSC) | ON248637 | 157,103 |
| *Calyptrocalyx forbesii* (Ridl.) Dowe & M.D.Ferrero | N_XT090 (IBSC) | ON248638 | 158,733 |
| *Calyptronoma rivalis* (O.F. Cook) L.H. Bailey | M_MD04 (IBSC) | ON248741 | 158,973 |
| *Carpentaria acuminata* (H. Wendl. & Drude) Becc. | M_XM016 (IBSC) | ON248742 | 156,862 |
| *Carpoxylon macrospermum* H. Wendl. & Drude | N_XT052 (IBSC) | ON248639 | 159,031 |
| *Carpoxylon macrospermum* H. Wendl. & Drude | M_XT195 (IBSC) | ON248743 | 159,014 |
| *Caryota obtusa* Griff. | M_SC872 (IBSC) | ON248744 | 159,514 |
| *Ceroxylon ventricosum* Burret | N_18121 (IBSC) | ON248640 | 158,545 |
| *Chamaedorea cataractarum* Mart. | N_SC019 (IBSC) | ON248641 | 156,988 |
| *Chamaedorea costaricana* Oerst. | N_XM044 (IBSC) | ON248642 | 157,002 |
| *Chamaedorea linearis* (Ruiz & Pav.) Mart. | M_18122 (IBSC) | ON248745 | 155,925 |
| *Chamaedorea seifrizii* Burret | N_XT030 (IBSC) | ON248643 | 156,897 |
| *Chamaedorea sp*_1 | M_zhpu90 (IBSC) | ON248746 | 156,533 |
| *Chamaedorea sp*_2 | N_18123 (IBSC) | ON248800 | 157,235 |
| *Chamaerops humilis* L. | M_XM060 (IBSC) | ON248747 | 158,646 |
| *Chambeyronia macrocarpa* Vieill. ex Becc. | M_SC074 (IBSC) | ON248748 | 158,882 |
| *Chrysalidocarpus baronii* Becc. | N_SC139 (IBSC) | ON248651 | 158,936 |
| *Chrysalidocarpus decaryi* (Jum.) Eiserhardt & W.J.Baker | N_SC888 (IBSC) | ON248652 | 159,530 |
| *Chrysalidocarpus leptocheilos* (Hodel) Eiserhardt & W.J. Baker | N_SC880 (IBSC) | ON248653 | 158,617 |
| *Chuniophoenix hainanensis* Burret | M_SC987 (IBSC) | ON248644 | 153,805 |
| *Chuniophoenix nana* Burret | N_SC963 (IBSC) | ON248645 | 155,340 |
| *Chuniophoenix suoitienensis* Henderson | M_ST12 (IBSC) | ON248749 | 153,584 |
| *Coccothrinax crinita* (Griseb. & H. Wendl. ex Kerch.) Becc. | M_XM402 (IBSC) | ON248750 | 158,180 |
| *Cocos nucifera* L. | N_XM116 (IBSC) | ON248646 | 155,012 |
| *Copernicia baileyana* León | N_XT122 (IBSC) | ON248647 | 158,173 |
| *Cryosophila warscewiczii* (H. Wendl.) Bartlett | M_SC138 (IBSC) | ON248751 | 157,824 |
| *Cyphophoenix nucele* H.E. Moore | M_XM177 (IBSC) | ON248752 | 158,857 |
| *Cyrtostachys renda Blume* | M_XT100 (IBSC) | ON248753 | 158,431 |
| *Deckenia nobilis* H. Wendl. ex Seem. | M_MD05 (IBSC) | ON248754 | 158,701 |
| *Desmoncus orthacanthos* Mart. | N_XT022 (IBSC) | OM743240 | 156,655 |
| *Dictyosperma album* (Bory) H.L. Wendl. & Drude ex Scheff. | N_SC999 (IBSC) | ON248650 | 158,318 |
| *Dransfieldia micrantha* (Becc.) W.J. Baker & Zona | M_MD06 (IBSC) | ON248755 | 159,160 |
| *Dypsis pinnatifrons* Mart. | N_XT225 (IBSC) | OP966667 | 159,111 |
| *Elaeis guineensis* L. | M_SCBG886 (IBSC) | ON248756 | 155,602 |
| *Euterpe precatoria* Mart. | M_XT230 (IBSC) | ON248757 | 156,507 |
| *Euterpe precatoria* Mart. | M_XT232 (IBSC) | ON248758 | 156,366 |
| *Gaussia attenuata* (O.F. Cook) Becc. | N_XT226 (IBSC) | ON248654 | 157,489 |
| *Geonoma longivaginata* H. Wendl. ex Spruce | M_XT222 (IBSC) | ON248759 | 157,684 |
| *Guihaia argyrata* (S.K. Lee & F.N. Wei) S.K. Lee, F.N. Wei & J. Dransf. | M_ZH205B (IBSC) | ON248655 | 159,075 |
| *Guihaia grossifibrosa* (Gagnepain) J. Dransfield | N_SC105 (IBSC) | ON248760 | 159,645 |
| *Hemithrinax ekmaniana* Burret | M_MD08 (IBSC) | ON248761 | 158,118 |
| *Heterospathe elata* Scheff. | M_XT218 (IBSC) | ON248762 | 158,597 |
| *Heterospathe elata* Scheff. | XTBG231 (IBSC) | ON248763 | 158,426 |
| *Howea forsteriana* (F. Muell. & H. Wendl.) Becc. | M_SC040 (IBSC) | ON248764 | 158,537 |
| *Hydriastele microcarpa* (Scheff.) W.J. Baker & Loo | M_XT047 (IBSC) | ON248765 | 158,675 |
| *Hydriastele microspadix* (Warb. ex K. Schum. & Lauterb.) Burret | M_XT054 (IBSC) | ON248766 | 158,618 |
| *Hyophorbe lagenicaulis* (L.H. Bailey) H.E. Moore | N_XM224 (IBSC) | ON248656 | 157,677 |
| *Hyphaene coriacea* Gaertn. | M_XT120 (IBSC) | ON248767 | 158,174 |
| *Iguanura wallichiana* (Mart.) Becc. | M_XT089 (IBSC) | ON248768 | 156,698 |
| *Itaya amicorum* H.E. Moore | M_MD12 (IBSC) | ON248771 | 158,058 |
| *Johannesteijsmannia magnifica* J. Dransf. | M_XT016 (IBSC) | ON248772 | 156,729 |
| *Jubaea chilensis* (Molina) Baill. | N_XM203 (IBSC) | ON248657 | 154,756 |
| *Kentiopsis oliviformis* (Brongn. & Gris) Brongn. | M_XT087 (IBSC) | ON248773 | 158,882 |
| *Kerriodoxa elegans* J. Dransf. | M_SC081 (IBSC) | ON248774 | 153,586 |
| *Lanonia centralis* (A.J. Hend., N.K. Ban & N.Q. Dung) A.J. Hend. & C.D. Bacon | M_SC877 (IBSC) | ON248775 | 157,151 |
| *Latania loddigesii* Mart. | M_SC055 (IBSC) | ON248777 | 158,164 |
| *Licuala fordiana* Becc. | N_XM216 (IBSC) | ON248658 | 158,292 |
| *Licuala lauterbachii* Dammer & K. Schum. | N_XT104 (IBSC) | ON248659 | 157,900 |
| *Licuala peltata* Roxb. | N_SC912 (IBSC) | ON248778 | 157,824 |
| *Licuala ramsayi* (F. Muell.) Domin | N_SC008 (IBSC) | ON248660 | 158,228 |
| *Licuala ramsayi* (F. Muell.) Domin | XTBG109 (IBSC) | ON248779 | 156,950 |
| *Lanonia hainanensis* (A.J. Hend., L.X. Guo & Barfod) A.J. Hend. & C.D. Bacon | M_XT025 (IBSC) | ON248780 | 156,786 |
| *Livistona australis* (R. Br.) Mart. | M_SCBG942 (IBSC) | ON248781 | 158,346 |
| *Livistona sp* | N_XT117 (IBSC) | ON248661 | 158,530 |
| *Lytocaryum weddellianum* (H. Wendl.) Toledo | M_SC965 (IBSC) | ON248782 | 155,182 |
| *Manicaria saccifera* Gaertn. | N_MD09 (IBSC) | ON248662 | 158,913 |
| *Masoala madagascariensis* Jum. | N_XM217 (IBSC) | ON248663 | 158,564 |
| *Mauritia carana* Wallace | M_XT158 (IBSC) | ON248783 | 158,577 |
| *Nannorrhops ritchieana* H. Wendl. | M_XT175 (IBSC) | ON248664 | 158,039 |
| *Nannorrhops ritchieana* H. Wendl. | N_XM254 (IBSC) | ON248665 | 158,039 |
| *Nenga pumila* H. Wendl. ex Schaedtler | M_SC962 (IBSC) | ON248784 | 157,694 |
| *Neoveitchia storckii* (H. Wendl.) Becc. | M_MD11 (IBSC) | ON248785 | 158,824 |
| *Nephrosperma sp* | M_XT181 (IBSC) | ON248786 | 158,425 |
| *Nephrosperma van-houtteanum* (H. Wendl. ex Van Houtte) Balf. f. | M_XT103 (IBSC) | ON248788 | 158,487 |
| *Nephrosperma van-houtteanum* (H. Wendl. ex Van Houtte) Balf. f. | M_MD10 (IBSC) | ON248787 | 158,558 |
| *Normanbya normanbyi* (W. Hill) L.H. Bailey | M_SC046 (IBSC) | ON248789 | 157,044 |
| *Nypa fruticans* Wurmb | N_SSH001 (IBSC) | ON248666 | 159,344 |
| *Oncosperma tigillarium* (Jack) Ridl. | M_MD13 (IBSC) | ON248790 | 156,617 |
| *Oraniopsis appendiculata* (F.M. Bailey) J. Dransf., A.K. Irvine & N.W. Uhl | M_SC133 (IBSC) | ON248792 | 158,605 |
| *Phoenicophorium borsigianum* (K. Koch) Stuntz | M_MD18 (IBSC) | ON248793 | 158,452 |
| *Phoenix acaulis* Roxb. | N_XM084 (IBSC) | ON248667 | 158,156 |
| *Phoenix canariensis* Wildpret | N_XT115 (IBSC) | ON248794 | 158,557 |
| *Phoenix dactylifera* L. | N_SC056 (IBSC) | ON248668 | 158,556 |
| *Phoenix loureiroi* Kunth | N_XM429 (IBSC) | ON248669 | 158,204, |
| *Phoenix pusilla* Gaertn. | N_SC065 (IBSC) | ON248670 | 158,498 |
| *Phoenix pusilla* Gaertn. | N_XT010 (IBSC) | ON248671 | 158,474 |
| *Phoenix reclinata* Jacq. | N_SC057 (IBSC) | ON248672 | 158,479 |
| *Phoenix roebelenii* O'Brien | N_XT007 (IBSC) | ON248673 | 158,301 |
| *Phoenix rupicola* T. Anderson | N_XT113 (IBSC) | ON248674 | 158,588 |
| *Phoenix sp* | N_XT116 (IBSC) | ON248675 | 158,478 |
| *Phoenix sp* | M_XM227 (IBSC) | ON248769 | 158,400 |
| *Phoenix theophrasti* Greuter | N_XT112 (IBSC) | ON248676 | 158,429 |
| *Phytelephas aequatorialis* Spruce | N_XT172 (IBSC) | ON248677 | 159,050 |
| *Pinanga baviensis* Becc. | M_SC142 (IBSC) | ON248795 | 157,533 |
| *Pinanga baviensis* Becc. | M_XT036 (IBSC) | ON248796 | 157,730 |
| *Pinanga sylvestris* (Lour.) Hodel | M_XT026 (IBSC) | ON248797 | 157,692 |
| *Plectocomia elongata* Mart. ex Blume | N_XT077 (IBSC) | ON248678 | 158,964 |
| *Plectocomia elongata* Mart. ex Blume | M_MD14 (IBSC) | ON248798 | 157,290 |
| *Ponapea ledermanniana* Becc. | M_MD15 (IBSC) | ON248799 | 157,311 |
| *Prestoea acuminata var. montana* (Graham) A.J. Hend. & Galeano | M_MD16 (IBSC) | ON248801 | 157,635 |
| *Pritchardia pacifica* Seem. & H. Wendl. | N_XT015 (IBSC) | ON248679 | 157,661 |
| *Ptychococcus sp* | M_MD17 (IBSC) | ON248803 | 157,720 |
| *Ptychosperma macarthurii* (H. Wendl. ex H.J. Veitch) H. Wendl. ex Hook. f. | M_SC889 (IBSC) | ON248804 | 156,968 |
| *Raphia vinifera* P. Beauv. | N_SC949 (IBSC) | ON248822 | 155,219 |
| *Ravenea rivularis* Jum. & H. Perrier | N_SC029 (IBSC) | ON248681 | 158,661 |
| *Reinhardtia gracilis* (H. Wendl.) Drude ex Dammer | N_SC060 (IBSC) | ON248682 | 158,017 |
| *Reinhardtia sp* | N_XM043 (IBSC) | ON248683 | 157,955 |
| *Rhapidophyllum hystrix* (Pursh) H. Wendl. & Drude | M_XT163 (IBSC) | ON248805 | 157,649 |
| *Rhapis excelsa* (Thunb.) A. Henry | M_SC895 (IBSC) | ON248806 | 158,495 |
| *Rhopaloblaste augusta* (Kurz) H.E. Moore | M_XT088 (IBSC) | ON248684 | 157,736 |
| *Rhopaloblaste ceramica* (Miq.) Burret | M_XM163 (IBSC) | ON248807 | 157,725 |
| *Rhopaloblaste sp* | M_MD19 (IBSC) | ON248808 | 157,797 |
| *Roscheria melanochaetes* (H. Wendl.) H. Wendl. ex Balf. f. | N_XM205 (IBSC) | ON248685 | 158,733 |
| *Roystonea regia* (Kunth) O.F. Cook | M_SC136 (IBSC) | ON248809 | 1155,680 |
| *Sabal bermudana* L.H. Bailey | N_SC026 (IBSC) | ON248686 | 158,915 |
| *Sabal causiarum* (O.F. Cook) Becc. | N_SC027 (IBSC) | ON248687 | 158,897 |
| *Sabal dealbata* hort. ex Standl. | N_SC012 (IBSC) | ON248688 | 158,892 |
| *Sabal domingensis* Becc. | N_XT168 (IBSC) | ON248689 | 158,847 |
| *Sabal etonia* Swingle ex Nash | N_SC091 (IBSC) | ON248690 | 158,848 |
| *Sabal etonia* Swingle ex Nash | N_XM407 (IBSC) | ON248691 | 158,865 |
| *Sabal mauritiiformis* (H. Karst.) Griseb. & H. Wendl. | N_XT111 (IBSC) | ON248692 | 158,888 |
| *Sabal mexicana* Mart. | N_SC053 (IBSC) | ON248693 | 158,852 |
| *Sabal minor* (Jacq.) Pers. | M_SC096 (IBSC) | ON248694 | 157,826 |
| *Sabal minor* (Jacq.) Pers. | N_SC052 (IBSC) | ON248695 | 158,859 |
| *Sabal palmetto* (Walter) Lodd. ex Schult. & Schult. f. | M_SC092 (IBSC) | ON248696 | 157,826 |
| *Sabal palmetto* (Walter) Lodd. ex Schult. & Schult. f. | PSC035 (IBSC) | ON248697 | 158,868 |
| *Sabal palmetto* (Walter) Lodd. ex Schult. & Schult. f. | N_SC076 (IBSC) | ON248810 | 158,844 |
| *Sabal rosei* (O.F. Cook) Becc. | N_SC137 (IBSC) | ON248698 | 158,734 |
| *Sabal uresana* Trel. | N_XM157 (IBSC) | ON248699 | 158,784 |
| *Salacca wallichiana* Mart. | M_XT148 (IBSC) | ON248811 | 158,437 |
| *Saribus merrillii* (Becc.) Bacon & W.J.Baker | M_SCBG952 (IBSC) | ON248812 | 156,598 |
| *Satakentia liukiuensis* (Hatus.) H.E. Moore | M_XT236 (IBSC) | ON248813 | 158,850 |
| *Schippia concolor* Burret | M_SC945 (IBSC) | ON248814 | 158,256 |
| *Serenoa repens* (W. Bartram) Small | N_XM182 (IBSC) | ON248700 | 158,729 |
| *Syagrus coronata* (Mart.) Becc. | M_SC022 (IBSC) | ON248701 | 155,126 |
| *Synechanthus fibrosus* (H. Wendl.) H. Wendl. | N_MD20 (IBSC) | ON248702 | 157,564 |
| *Thrinax excelsa* Lodd. ex Mart. | M_SC063 (IBSC) | ON248815 | 158,046 |
| *Trachycarpus fortunei* (Hook.) H. Wendl. | N_XM057 (IBSC) | ON248703 | 158,743 |
| *Trachycarpus fortunei* (Hook.) H. Wendl. | N_XM214 (IBSC) | ON248704 | 158,696 |
| *Trachycarpus fortunei* (Hook.) H. Wendl. | M_XM107 (IBSC) | ON248816 | 157,719 |
| *Trachycarpus geminisectus* Spanner, Gibbons, V.D. Nguyen & T.P. Anh | M_SC988 (IBSC) | ON248817 | 159,018 |
| *Trachycarpus martianus* (Wall.) H. Wendl. | N_SC947 (IBSC) | ON248705 | 158,605 |
| *Trachycarpus nanus* Becc. | N_XT167 (IBSC) | ON248706 | 158,719 |
| *Trachycarpus oreophilus* Gibbons & Spanner | N_XT169 (IBSC) | ON248707 | 159,049 |
| *Trachycarpus princeps* Gibbons, Spanner & S.Y. Chen | N_XM226 (IBSC) | ON248708 | 158,708 |
| *Trithrinax campestris* (Burmeist.) Drude & Griseb. | M_SC080 (IBSC) | ON248818 | 158,351 |
| *Veitchia arecina* Becc. | N_XT042 (IBSC) | ON248709 | 158,407 |
| *Veitchia joannis* H. Wendl. | M_SC088 (IBSC) | ON248710 | 156,983 |
| *Veitchia subdisticha* (H.E. Moore) C. Lewis & Zona | M_XT233 (IBSC) | ON248770 | 156,965 |
| *Verschaffeltia splendida* H. Wendl. | M_XT102 (IBSC) | ON248819 | 158,664 |
| *Vonitra fibrosa* (C.H.Wright) Becc. | N_XT094 (IBSC) | OP952737 | 158,722 |
| *Wallichia caryotoides* Roxb. | N_XT063 (IBSC) | ON248711 | 160,075 |
| *Wallichia densiflora* Mart. | N_XT060 (IBSC) | ON248712 | 159,789 |
| *Wallichia disticha* T. Anderson | N_XM022 (IBSC) | ON248713 | 159,821 |
| *Wallichia disticha* T. Anderson | N_XM054 (IBSC) | ON248714 | 159,845 |
| *Wallichia gracilis* Becc. | N_SC016 (IBSC) | ON248715 | 159,700 |
| *Wallichia oblongifolia* Griff. | N_SC007_2 (IBSC) | ON248717 | 159,948 |
| *Wallichia oblongifolia* Griff. | N_SC007_1 (IBSC) | ON248716 | 159,996 |
| *Wallichia oblongifolia* Griff. | N_XM073 (IBSC) | ON248718 | 160,040 |
| *Washingtonia filifera* (Gloner ex Kerch., Burv., Pynaert, Rodigas & Hull) de Bary | N_SC881 (IBSC) | ON248719 | 157,924 |
| *Wodyetia bifurcata* A.K. Irvine | N_SC034 (IBSC) | ON248720 | 159,033 |
| *Zombia antillarum* (Descourt.) L.H. Bailey | M_XT216 (IBSC) | ON248820 | 157,839 |
| *Zombia antillarum* (Descourt.) L.H. Bailey | M_MD07 (IBSC) | ON248821 | 157,502 |
| *Acoelorraphe wrightii* (Griseb. & H. Wendl.) H. Wendl. ex Becc. |  | NC_029973 ✱ |  |
| *Acrocomia aculeata* (Jacq.) Lodd. ex Mart. |  | NC_037084 ✱ |  |
| *Areca vestiaria* Giseke |  | KP221698 ✱ |  |
| *Areca vestiaria* Giseke |  | NC_029972 ✱ |  |
| *Arenga caudata* (Lour.) H.E. Moore |  | NC_029971 ✱ |  |
| *Arenga pinnata* (Wurmb) Merr. |  | NC_045907 ✱ |  |
| *Astrocaryum aculeatum* G. Mey. |  | MH537788 ✱ |  |
| *Astrocaryum murumuru* Mart. |  | MH537787 ✱ |  |
| *Attalea speciosa* Mart. |  | KP221699 ✱ |  |
| *Bactris major* Jacq. |  | KP221700 ✱ |  |
| *Beccariophoenix madagascariensis* Jum. & H. Perrier |  | KP221701 ✱ |  |
| *Bismarckia nobilis* Hildebrandt & H. Wendl. |  | NC_020366 ✱ |  |
| *Borassodendron machadonis* (Ridl.) Becc. |  | NC_029969 ✱ |  |
| *Borassus flabellifer* L. |  | KP901247 ✱ |  |
| *Brahea brandegeei* (Purpus) H.E. Moore |  | NC_029968 ✱ |  |
| *Burretiokentia grandiflora* Pintaud & Hodel |  | KP221702 ✱ |  |
| *Calamus caryotoides* A. Cunn. ex Mart. |  | NC_020365 ✱ |  |
| *Caryota mitis* Lour. |  | NC_029948 ✱ |  |
| *Chamaedorea seifrizii* Burret |  | JX088667 ✱ |  |
| *Chrysalidocarpus decaryi* (Jum.) Eiserhardt & W.J. Baker |  | KP221705 ✱ |  |
| *Chuniophoenix nana* Burret |  | NC_029966 ✱ |  |
| *Cocos nucifera* L. |  | NC_022417 ✱ |  |
| *Colpothrinax cookii* Read |  | NC_028026 ✱ |  |
| *Corypha lecomtei* Becc. ex Lecomte |  | NC_029965 ✱ |  |
| *Dictyosperma album* (Bory) H.L. Wendl. & Drude ex Scheff. |  | KP221703 ✱ |  |
| *Drymophloeus litigiosus* (Becc.) H.E. Moore |  | KP221704 ✱ |  |
| *Elaeis guineensis* Jacq. |  | NC_017602 ✱ |  |
| *Eremospatha macrocarpa* G. Mann & H. Wendl. ex Schaedtler |  | NC_029964 ✱ |  |
| *Eugeissona tristis* Griff. |  | NC_029963 ✱ |  |
| *Geonoma undata subsp. dussiana* (Becc.) A.J. Hend. |  | KP221706 ✱ |  |
| *Chamaerops humilis* Klotzsch |  | NC_029967 ✱ |  |
| *Heterospathe cagayanensis* Becc. |  | KP221707 ✱ |  |
| *Hydriastele microspadix* (Warb. ex K. Schum. & Lauterb.) Burret |  | KP221708 ✱ |  |
| *Iriartea deltoidea* Ruiz & Pav. |  | KP221709 ✱ |  |
| *Kentiopsis piersoniorum* Pintaud & Hodel |  | KP221710 ✱ |  |
| *Leopoldinia pulchra* Mart. |  | KP221711 ✱ |  |
| *Leucothrinax morrisii* (H. Wendl.) C. Lewis & Zona |  | NC_029961 ✱ |  |
| *Licuala paludosa* Griff. |  | KT312928 ✱ |  |
| *Lodoicea maldivica* (J.F. Gmel.) Pers. ex H. Wendl. |  | NC_029960 ✱ |  |
| *Manicaria saccifera* Gaertn. |  | KP221712 ✱ |  |
| *Mauritia flexuosa* L. f. |  | NC_029947 ✱ |  |
| *Metroxylon warburgii* Becc. |  | NC_029959 ✱ |  |
| *Nypa fruticans* Wurmb |  | NC_029958 ✱ |  |
| *Oenocarpus bataua* Mart. |  | KP221713 ✱ |  |
| *Oenocarpus minor* Mart. |  | KP221714 ✱ |  |
| *Orania palindan* (Blanco) Merr. |  | KP221686 ✱ |  |
| *Pelagodoxa henryana* Becc. |  | KP221687 ✱ |  |
| *Phoenix dactylifera* L. |  | FJ212316 ✱ |  |
| *Phoenix dactylifera* L. |  | MF176947 ✱ |  |
| *Phoenix dactylifera* L. |  | MF197494 ✱ |  |
| *Phoenix dactylifera* L. |  | MF197495 ✱ |  |
| *Phoenix dactylifera* L. |  | NC_013991 ✱ |  |
| *Phytelephas aequatorialis* Spruce |  | NC_029957 ✱ |  |
| *Pigafetta elata* (Mart.) H. Wendl. |  | NC_029956 ✱ |  |
| *Podococcus barteri* Mann & Wendland |  | NC_027276 ✱ |  |
| *Prestoea acuminata var. montana* (Graham) A.J. Hend. & Galeano |  | KP221689 ✱ |  |
| *Pritchardia thurstonii* F. Muell. & Drude |  | NC_029955 ✱ |  |
| *Pseudophoenix vinifera* (Mart.) Becc. |  | NC_020364 ✱ |  |
| *Archontophoenix alexandrae* (F. Muell.) H. Wendl. & Drude |  | NC 046017 ✱ |  |
| *Reinhardtia gracilis* H. Wendl.) Drude ex Dammer |  | KP221690 ✱ |  |
| *Reinhardtia latisecta* (H. Wendl.) Burret |  | KP221691 ✱ |  |
| *Reinhardtia paiewonskiana* Read, Zanoni & M.M. Mejía |  | KP221693 ✱ |  |
| *Reinhardtia simplex* (H. Wendl.) Drude ex Dammer |  | KP221694 ✱ |  |
| *Roystonea regia* (Kunth) O.F. Cook |  | KP221692 ✱ |  |
| *Sabal domingensis* Becc. |  | NC_026444 ✱ |  |
| *Salacca ramosiana* Mogea |  | NC_029954 ✱ |  |
| *Satakentia liukiuensis* (Hatus.) H.E. Moore |  | KP221695 ✱ |  |
| *Sclerosperma profizianum* Valk. & Sunderl. |  | KP221696 ✱ |  |
| *Serenoa repens* (W. Bartram) Small |  | NC_029953 ✱ |  |
| *Syagrus coronata* (Mart.) Becc. |  | NC_029241 ✱ |  |
| *Tahina spectabilis* J. Dransf. & Rakotoarinivo |  | NC_029952 ✱ |  |
| *Trithrinax brasiliensis* Mart. |  | NC_029951 ✱ |  |
| *Veitchia arecina* Becc. |  | NC_029950 ✱ |  |
| *Veitchia spiralis* H. Wendl. |  | KP221697 ✱ |  |
| *Wallichia densiflora* Mart. |  | NC_029949 ✱ |  |
| *Washingtonia robusta* H. Wendl. |  | NC_029974 ✱ |  |
|  |  |  |  |
| **Outgroups** |  |  |  |
| *Acorus americanus* (Raf.) Raf. |  | NC_010093 ✱ |  |
| *Alstroemeria aurea* Graham |  | KC968976 ✱ |  |
| *Baxteria australis* R. Br. ex Hook. |  | NC_029970 ✱ |  |
| *Calectasia narragara* R.L. Barrett & K.W. Dixon |  | JX088666 ✱ |  |
| *Colocasia esculenta* (L.) Schott |  | KY769273 ✱ |  |
| *Dasypogon bromeliifolius* R. Br. |  | NC_020367 ✱ |  |
| *Dioscorea aspersa* Prain & Burkill |  | NC_039807 ✱ |  |
| *Eichhornia crassipes* (Mart.) Solms |  | NC_046773 ✱ |  |
| *Japonolirion osense* Nakai |  | NC_036154 ✱ |  |
| *Kingia australis* R. Br. |  | JX051651 ✱ |  |
| *Oryza sativa* L. |  | MK348618 ✱ |  |
| *Pandanus tectorius* Parkinson |  | NC_042747 ✱ |  |
| *Polygonatum cyrtonema* Hua |  | NC_028429 ✱ |  |
| *Zingiber officinale* Roscoe |  | NC_044775 ✱ |  |
