# Supplementary material for: A plastid phylogenomic framework for the palm family (Arecaceae)
Source: BMC Biol. 2023 Mar 8;21:50. doi: 10.1186/s12915-023-01544-y (PMC9993706; doi:10.1186/s12915-023-01544-y)
Supplement: Supplementary file 2 — Additional file 2: Table S2. Accessions of the 49 genera with few plastid DNA regions available and therefore a large proportion of missing data in the incomplete-105 regions matrix, with GenBank accession numbers of sequences used in the present study. The character ‘—’ indicates that the sequence was unavailable from NCBI (https://www.ncbi.nlm.nih.gov/) and treated as missing data in the matrix. [file 12915_2023_1544_MOESM2_ESM.docx]

**Table S2** Accessions of the 49 genera with few plastid DNA regions available and therefore a large proportion of missing data in the incomplete-105 regions matrix, with GenBank accession numbers of sequences used in the present study. The character ‘—’ indicates that the sequence was unavailable from NCBI (https://www.ncbi.nlm.nih.gov/) and treated as missing data in the matrix.

|  | ***trnD-trnT*** | ***rps16* intron** | ***rbcL*** | ***ndhF*** | ***matK*** | ***rpoC1*** | ***accD*** | ***trnL-F*** | ***trnQ-rps16*** | ***atpB*** | ***atpF*** | ***ndhG*** | ***ndhA* intron** | ***rps15-ycf1*** |
| --- | --- | --- | --- | --- | --- | --- | --- | --- | --- | --- | --- | --- | --- | --- |
| ***Acanthophoenix*** | — | MG647542 | MG437726 | MG647281 | AM114691 | MG438236 | — | AM113679 | — | — | — | — | — | — |
| ***Adonidia*** | — | MG647546 | MG437730 | MG647285 | MK704941 | MG438239 | MG437986 | AB817688 | — | — | — | — | KJ598474 | KJ598320 |
| ***Ammandra*** | — | MG647425 | MG437610 | MG647166 | EF128232 | MG438125 | MG437871 | AJ404922 | — | — | — | — | — | — |
| ***Aphandra*** | — | MG647426 | MG437611 | MG647167 | EF128233 | MG438126 | MG437872 | — | EF605533 | AY044458 | — | — | — | — |
| ***Balaka*** | — | MG647549 | MG437733 | MG647288 | KJ598360 | MG438242 | MG437989 | AJ241305 | — | AY012436 | — | — | KJ598457 | KJ598304 |
| ***Barcella*** | — | MG647476 | MG437660 | MG647217 | HQ265562 | MG438173 | MG437920 | HQ265797 | HQ265466 | AY044467 | — | — | — | — |
| ***Calyptrogyne*** | MK102100 | AJ238816 | MG437673 | MG647230 | AM114652 | MG438185 | MG437932 | AM113655 | — | — | — | — | — | — |
| ***Chelyocarpus*** | — | MG647357 | MG437542 | MG647098 | KY020630 | MG438058 | MG437804 | AJ241254 | — | — | — | — | — | — |
| ***Clinosperma*** | — | MG647522 | MG437706 | MG647263 | AM114680 | MG438217 | MG437964 | AM113674 | EF605584 | — | — | — | — | — |
| ***Clinostigma*** | AF449162 | MG647564 | MG437748 | MG647303 | AM114706 | MG438257 | MG438004 | AM113688 | AF449148 | AF449171 | — | — | — | — |
| ***Cyphokentia*** | — | MG647521 | MG437705 | MG647262 | AM114677 | MG438216 | MG437963 | AM113671 | EF605583 | — | — | — | — | — |
| ***Cyphosperma*** | — | HG969822 | MG437698 | MG647255 | HG969988 | MG438210 | MG437957 | HG969954 | HG969921 | — | — | — | — | — |
| ***Dictyocaryum*** | — | MG647430 | MG437615 | MG647171 | AM114616 | MG438129 | MG437875 | AM113640 | — | AY012422 | — | — | KF775967 | — |
| ***Eleiodoxa*** | — | MG647333 | AJ829868 | EU186185 | — | MG438034 | MG437779 | — | — | — | — | — | — | — |
| ***Hedyscepe*** | — | MG647558 | MG437742 | MG647297 | AM114702 | MG438251 | MG437998 | AJ404905 | — | — | — | — | KJ598483 | KJ598330 |
| ***Hyospathe*** | — | MG647481 | MG437665 | MG647222 | AM114646 | MG438178 | MG437925 | AJ241300 | EF605568 | — | — | — | — | — |
| ***Iriartella*** | — | MG647429 | MG437614 | MG647170 | AM114615 | MG438128 | MG437874 | AM113639 | — | — | — | — | KF775962 | — |
| ***Juania*** | — | MG647421 | MG437606 | MG647162 | AM114608 | MG438121 | MG437867 | AM113638 | KM597759 | — | KP255439 | KJ170927 | — | — |
| ***Jubaeopsis*** | HQ265754 | HQ265708 | MG437641 | EU004879 | AM114633 | MG438155 | MG437902 | HQ265803 | EF605534 | — | — | — | — | — |
| ***Korthalsia*** | EU117431 | MG647332 | MG437516 | MG647074 | AM114546 | MG438033 | MG437778 | AM113613 | — | — | — | — | — | — |
| ***Laccospadix*** | — | AJ240895 | AJ404812 | MG647278 | AM114689 | MG438233 | MG437981 | AJ241304 | — | — | — | — | — | — |
| ***Laccosperma*** | EU117424 | MG647327 | AJ404772 | EU186179 | AM114543 | MG438028 | MG437773 | AJ241276 | — | — | — | — | — | — |
| ***Lemurophoenix*** | — | MG647526 | MG437710 | MG647267 | AM114682 | MG438221 | MG437968 | AJ404902 | — | — | — | — | — | — |
| ***Lepidocaryum*** | EU117457 | MG647329 | AJ829880 | EU186210 | — | MG438030 | MG437775 | — | — | — | — | — | — | — |
| ***Lepidorrhachis*** | — | AM116850 | MG437699 | MG647256 | AM114715 | MG438211 | MG437958 | AM113693 | — | — | — | — | — | — |
| ***Linospadix*** | DQ227239 | MG647537 | AJ404811 | AF449144 | AM114688 | MG438231 | MG437979 | KC428547 | EF605586 | AF449172 | — | — | — | — |
| ***Loxococcus*** | — | MG647573 | MG437757 | MG647312 | AM114716 | MG438266 | MG438013 | AM113694 | — | — | — | — | — | KJ598326 |
| ***Manjekia*** | — | MG647547 | MG437731 | MG647286 | KJ598374 | MG438240 | MG437987 | — | — | — | — | — | KJ598477 | KJ598323 |
| ***Marojejya*** | — | MG647529 | MG437713 | MG647270 | AM114684 | MG438224 | MG437971 | AM113675 | — | — | — | — | — | — |
| ***Mauritiella*** | — | MG647331 | MG437515 | MG647073 | FR832790 | MG438032 | MG437777 | — | — | — | — | — | — | — |
| ***Maxburretia*** | HQ720446 | MG647370 | MG437555 | MG647111 | HQ720297 | MG438070 | MG437817 | AM113624 | — | — | — | — | — | — |
| ***Medemia*** | AM903277 | MG647410 | AJ829885 | MG647151 | AM114600 | MG438110 | MG437856 | AM113635 | — | — | — | — | — | — |
| ***Myrialepis*** | EU117448 | MG647338 | MG437522 | EU186201 | MK984750 | MG438038 | MG437784 | — | — | — | — | — | — | — |
| ***Neonicholsonia*** | EU004860 | AJ240890 | MG437668 | MG647225 | AM114649 | MG438181 | MG437928 | AJ241299 | EF605570 | — | — | — | — | — |
| ***Oncocalamus*** | EU117455 | MG647325 | MG437509 | MG647067 | AM114541 | MG438026 | MG437771 | — | — | — | — | — | — | — |
| ***Parajubaea*** | EU004231 | MG647466 | MG437650 | MG647207 | — | MG438164 | MG437911 | — | — | — | — | — | — | — |
| ***Pholidocarpus*** | HQ720405 | MG647377 | MG437562 | MG647118 | HQ720295 | MG438077 | MG437824 | AM113625 | — | — | — | — | — | — |
| ***Pholidostachys*** | MK102135 | MG647487 | MG437671 | MG647228 | AM114651 | — | — | AM113654 | — | — | — | — | — | — |
| ***Physokentia*** | — | MG647516 | MG437700 | MG647257 | AM114671 | MG438212 | MG437959 | AM113665 | — | — | — | — | — | — |
| ***Plectocomiopsis*** | EU117449 | — | AJ829900 | EU186202 | — | MG438039 | MG437785 | — | — | — | — | — | — | — |
| ***Rhopalostylis*** | — | MG647557 | MG437741 | MG647296 | HG969985 | MG438250 | MG437997 | HG969951 | HG969918 | — | — | — | KJ598484 | KJ598331 |
| ***Satranala*** | AM903278 | MG647407 | AJ404771 | MG647148 | AM114598 | MG438107 | MG437853 | AJ241275 | — | — | — | — | — | — |
| ***Socratea*** | KF775745 | MG647432 | MG437617 | EU004875 | AM114618 | MG438131 | MG437877 | AM113641 | — | AY012423 | — | — | KF775970 | — |
| ***Sommieria*** | — | MG647499 | MG437683 | MG647240 | AM114658 | MG438195 | MG437942 | AM113657 | — | — | — | — | — | — |
| ***Tectiphiala*** | — | MG647543 | MG437727 | MG647282 | AM114692 | — | — | AM113680 | — | — | — | — | — | — |
| ***Voanioala*** | HQ265758 | HQ265712 | AY044635 | AY044569 | HQ265573 | — | — | HQ265807 | HQ265477 | AY044472 | — | — | — | — |
| ***Welfia*** | MK102150 | AM116809 | MG437670 | MG647227 | AM114650 | MG438183 | MG437930 | AM113653 | — | — | — | — | — | — |
| ***Wendlandiella*** | AY044492 | MG647435 | AY012477 | AY044541 | DQ178701 | MG438134 | MG437880 | AM113642 | — | AY012420 | — | — | — | — |
| ***Wettinia*** | KF775715 | MG647433 | MG437618 | EU004876 | AM114619 | MG438132 | MG437878 | AJ404898 | AY044593 | AY012424 | — | — | KF775981 | — |
