# Supplementary material for: A plastid phylogenomic framework for the palm family (Arecaceae)
Source: BMC Biol. 2023 Mar 8;21:50. doi: 10.1186/s12915-023-01544-y (PMC9993706; doi:10.1186/s12915-023-01544-y)
Supplement: Supplementary file 3 — Additional file 3: Table S3. Proportion of missing data for the 49 accessions (genera) with few DNA sequences available, compared to the total length of the incomplete-105 regions matrix, and bootstrap value for the stem nodes of these accessions (genera) obtained from maximum likelihood (ML) analysis of the incomplete-105 regions matrix (Fig. 1). [file 12915_2023_1544_MOESM3_ESM.docx]

**Tables S3** Proportion of missing data for the 49 accessions (genera) with few DNA sequences available, compared to the total length of the incomplete-105 regions matrix, and bootstrap value for the stem nodes of these accessions (genera) obtained from maximum likelihood (ML) analysis of the incomplete-105 regions matrix (Fig. 1).

| **Accessions (Genera)** | **Proportion of missing data in the incomplete-105 regions matrix** | **Bootstrap value for the stem node of the genus** |
| --- | --- | --- |
| *Acanthophoenix* H. Wendl. | 93.46% | 96% |
| *Adonidia* Becc. | 92.65% | 66% |
| *Ammandra* O.F. Cook | 92.05% | 60% |
| *Aphandra* Barfod | 91.39% | 100% |
| *Balaka* Becc. | 89.80% | 100% |
| *Barcella* Trail ex Drude | 89.23% | 99% |
| *Calyptrogyne* H. Wendl. | 92.17% | 100% |
| *Chelyocarpus* Dammer | 92.13% | 54% |
| *Clinosperma* Becc. | 91.78% | 74% |
| *Clinostigma* H. Wendl. | 89.43% | 59% |
| *Cyphokentia* Brongn. | 91.79% | 74% |
| *Cyphosperma* H. Wendland ex J. E. Hooker in Benth. & Hook. f. | 91.71% | < 50% |
| *Dictyocaryum* H. Wendl. | 89.36% | 62% |
| *Eleiodoxa* Burret | 94.82% | 67% |
| *Hedyscepe* H. Wendl. & Drude | 91.33% | 83% |
| *Hyospathe* Mart. | 90.80% | < 50% |
| *Iriartella* H. Wendl. | 90.95% | 62% |
| *Juania* Drude | 91.10% | 100% |
| *Jubaeopsis* Becc. | 91.10% | 79% |
| *Korthalsia* Blume | 92.17% | 91% |
| *Laccospadix* Drude & H. Wendland | 92.96% | 64% |
| *Laccosperma* Drude | 92.08% | 100% |
| *Lemurophoenix* J. Dransf. | 92.97% | 73% |
| *Lepidocaryum* Mart. | 94.82% | 75% |
| *Lepidorrhachis* O.F. Cook | 93.12% | 79% |
| *Linospadix* H. Wendland in H. Wendland & Drude | 88.22% | 64% |
| *Loxococcus* H. Wendl. & Drude | 92.69% | < 50% |
| *Manjekia* W.J. Baker & Heatubun | 92.46% | < 50% |
| *Marojejya* Humbert | 93.13% | 70% |
| *Mauritiella* Burret | 94.80% | 75% |
| *Maxburretia* Furtado | 92.21% | 52% |
| *Medemia* Wurttemb. ex H. Wendl. | 92.23% | 95% |
| *Myrialepis* Becc. | 93.81% | 89% |
| *Neonicholsonia* Dammer | 90.72% | < 50% |
| *Oncocalamus* (G. Mann & H. Wendl.) H. Wendl. | 93.17% | 76% |
| *Parajubaea* Burret | 94.79% | < 50% |
| *Pholidocarpus* Blume | 92.67% | < 50% |
| *Pholidostachys* H. Wendl. ex Hook. f. | 93.16% | 76% |
| *Physokentia* Becc. | 93.14% | < 50% |
| *Plectocomiopsis* Becc. | 95.82% | 62% |
| *Rhopalostylis* Klotzsch ex Baill. | 90.09% | 83% |
| *Satranala* J. Dransf. & Beentje | 92.06% | 99% |
| *Socratea* H. Karst. | 89.38% | 72% |
| *Sommieria* Becc. | 93.08% | 100% |
| *Tectiphiala* H.E. Moore | 94.05% | 96% |
| *Voanioala* J. Dransf. | 89.41% | 79% |
| *Welfia* H. Wendl. | 92.22% | 76% |
| *Wendlandiella* Dammer | 89.49% | 51% |
| *Wettinia* Poepp. | 87.90% | 72% |
