# Supplementary figures and images for: A plastid phylogenomic framework for the palm family (Arecaceae)
Source: BMC Biol. 2023 Mar 8;21:50. doi: 10.1186/s12915-023-01544-y (PMC9993706; doi:10.1186/s12915-023-01544-y)

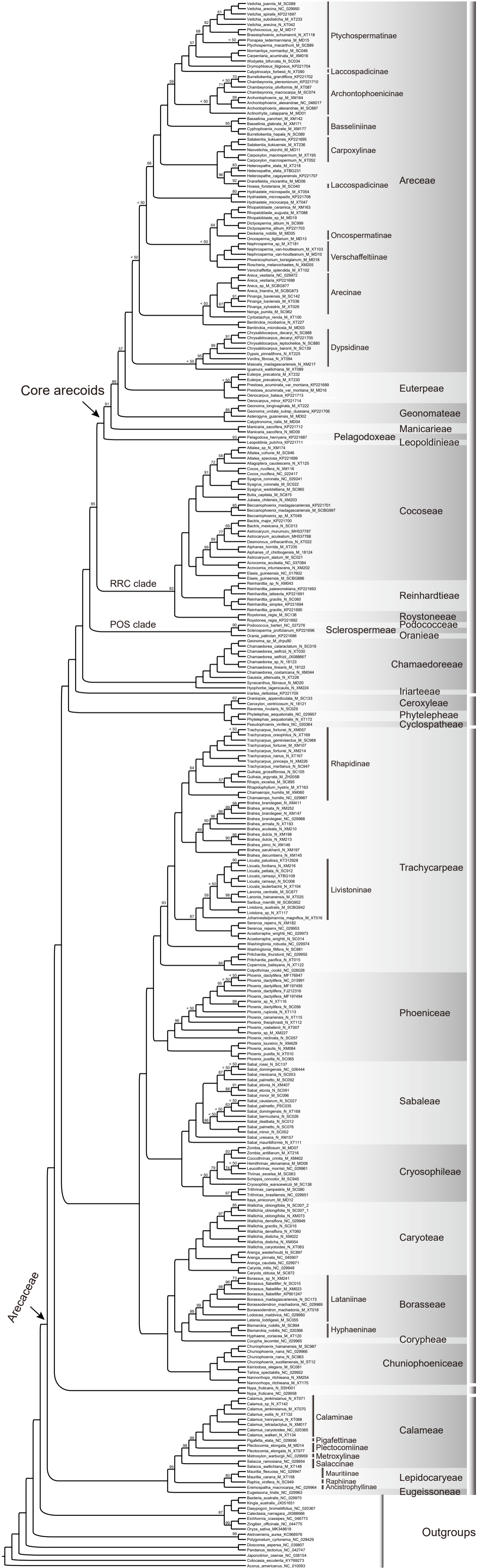

Arecoideae

Ceroxyloideae

Coryphoideae

Calamoideae

Outgroups

Supplement: Supplementary file 4 — Additional file 4: Fig. S1. Maximum likelihood phylogenetic tree of Arecaceae inferred from the complete-coding matrix. Bootstrap values inferior to 100% are shown, with dashes denoting a support inferior to 50%. [file 12915_2023_1544_MOESM4_ESM.pdf]

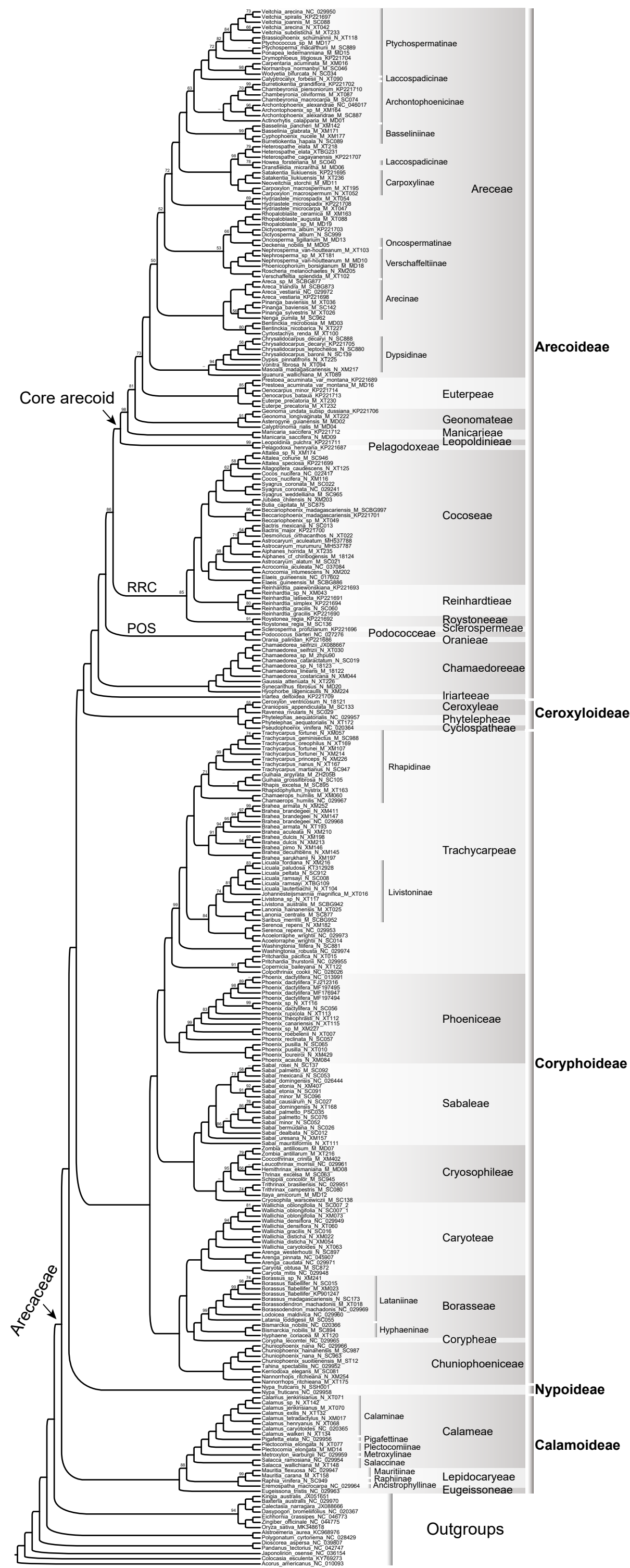

Supplement: Supplementary file 5 — Additional file 5: Fig. S2. Maximum likelihood phylogenetic tree of Arecaceae inferred from the complete-105 regions matrix. Bootstrap values inferior to 100% are shown, with dashes denoting a support inferior to 50%. [file 12915_2023_1544_MOESM5_ESM.pdf]
