# Supplementary material for: A plastid phylogenomic framework for the palm family (Arecaceae)
Source: BMC Biol. 2023 Mar 8;21:50. doi: 10.1186/s12915-023-01544-y (PMC9993706; doi:10.1186/s12915-023-01544-y)
Supplement: Supplementary file 6 — Additional file 6: Fig. S3. Maximum likelihood phylogenetic tree (including branch lengths) of Arecaceae inferred from the incomplete-105 regions matrix. [file 12915_2023_1544_MOESM6_ESM.pdf]

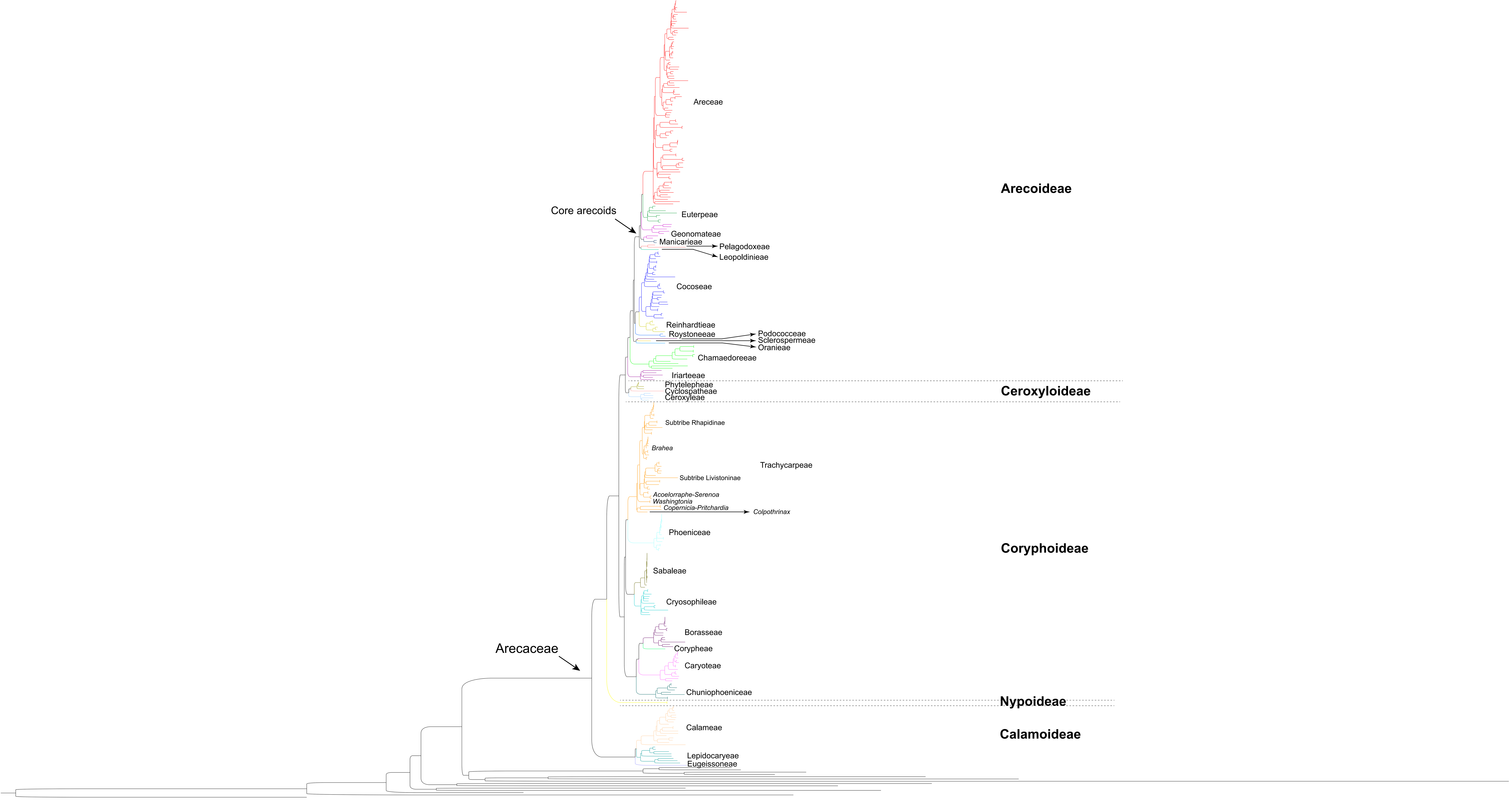

Areceae
Arecoideae
Core arecoids
Euterpeae
Geonomateae
Manicarieae
Pelagodoxeae
Leopoldinieae
Cocoseae
Reinhardtieae
Roystoneeae
Podococceae
Sclerospermeae
Oranieae
Chamaedoreeae
Iriarteeae
Phytelepheae
Cyclospatheae
Ceroxyleae
Ceroxyloideae
Subtribe Rhapidinae
Brahea
Trachycarpeae
Subtribe Livistoninae
Acoelorraphe-Serenoa
Washingtonia
Copernicia-Pritchardia
Colpothrinax
Phoeniceae
Coryphoideae
Sabaleae
Cryosophileae
Borasseae
Corypheae
Arecaceae
Caryoteae
Chuniophoeniceae
Nypoideae
Calameae
Calamoideae
Lepidocaryeae
Eugeissoneae
